# Social Data: Biases, Methodological Pitfalls, and Ethical Boundaries

**DOI:** 10.3389/fdata.2019.00013

**Published:** 2019-07-11

**Authors:** Alexandra Olteanu, Carlos Castillo, Fernando Diaz, Emre Kıcıman

**Affiliations:** ^1^Microsoft Research, New York, NY, United States; ^2^Microsoft Research, Montreal, QC, Canada; ^3^Department of Information and Communication Technologies, Universitat Pompeu Fabra, Barcelona, Spain; ^4^Microsoft Research, Redmond, WA, United States

**Keywords:** social media, user data, biases, evaluation, ethics

## Abstract

Social data in digital form—including user-generated content, expressed or implicit relations between people, and behavioral traces—are at the core of popular applications and platforms, driving the research agenda of many researchers. The promises of social data are many, including understanding “what the world thinks” about a social issue, brand, celebrity, or other entity, as well as enabling better decision-making in a variety of fields including public policy, healthcare, and economics. Many academics and practitioners have warned against the naïve usage of social data. There are biases and inaccuracies occurring at the source of the data, but also introduced during processing. There are methodological limitations and pitfalls, as well as ethical boundaries and unexpected consequences that are often overlooked. This paper recognizes the rigor with which these issues are addressed by different researchers varies across a wide range. We identify a variety of menaces in the practices around social data use, and organize them in a framework that helps to identify them.

“*For your own sanity, you have to remember that not all problems can be solved. Not all problems can be solved, but all problems can be illuminated.” –Ursula Franklin*^1^

## 1. Introduction

We use *social data* as an umbrella concept for all kind of digital traces produced by or about users, with an emphasis on content explicitly written with the intent of communicating or interacting with others. Social data typically comes from *social software*, which provides an intermediary or a focus for a social relationship (Schuler, 1994). It includes a variety of *platforms*—like for social media and networking (e.g., Facebook), question and answering (e.g., Quora), or collaboration (e.g., Wikipedia)—and *purposes* from finding information (White, 2013) to keeping in touch with friends (Lampe et al., 2008). Social software enables the *social web*, a class of websites “in which user participation is the primary driver of value” (Gruber, 2008).

The social web enables access to social traces at a scale and level of detail, both in breadth and depth, impractical with conventional data collection techniques, like surveys or user studies (Richardson, 2008; Lazer et al., 2009). On the social web users search, interact, and share information on a mix of topics including work (Ehrlich and Shami, 2010), food (Abbar et al., 2015), or health (De Choudhury et al., 2014); leaving, as a result, rich traces that form what Harford (2014) calls *found data*: “the digital exhaust of web searches, credit card payments and mobiles pinging the nearest phone mast.”

People provide these data for many reasons: these platforms allow them to achieve some goals or receive certain benefits. Motivations include communication, friendship maintenance, job seeking, or self-presentation (Lampe et al., 2006; Joinson, 2008), which are often also key to understanding ethical facets of social data use.

Social data opens unprecedented opportunities to answer significant questions about society, policies, and health, being recognized as one core reason behind progress in many areas of computing (e.g., crisis informatics, digital health, computational social science) (Crawford and Finn, 2014; Tufekci, 2014; Yom-Tov, 2016). They are believed to provide insights into both individual-level and large human phenomena, with a plethora of applications and substantial impact (Lazer et al., 2009; Dumais et al., 2014; Harford, 2014; Tufekci, 2014). Concomitantly, there is also a growing consensus that while the ever-growing datasets of online social traces offer captivating insights, they are *more than just an observational tool*.

### 1.1. A Growing Concern

In this paper we aim to strengthen prior calls—including boyd and Crawford (2012); Ruths and Pfeffer (2014); Tufekci (2014); Ekbia et al. (2015) and Gillespie (2015)—to carefully scrutinize the use of social data against a variety of possible data and methodological pitfalls. Social data are being leveraged to make inferences about how much to pay for a product (Hannak et al., 2014), about the likelihood of being a terrorist or about users health (Yom-Tov, 2016) and employability (Rosenblat et al., 2014).^2^ While such inferences are increasingly used in decision- and policy-making, they can also have important negative implications (Diakopoulos, 2016; O'Neil, 2016). Yet, such implications are not always well understood or recognized (Tufekci, 2014; O'Neil, 2016), as many seem to assume that these data, and the frameworks used to handle them, are *adequate*, often *as-is*, for the problem at hand, with little or no scrutiny. A key concern is that research agendas tend to be opportunistically driven by access to data, tools, or ease of analysis (Ruths and Pfeffer, 2014; Tufekci, 2014; Weller and Gorman, 2015); or, as Baeza-Yates (2013) puts it, “we see a lot of data mining for the sake of it.”

In the light of Google Flu Trends' initial success (Ginsberg et al., 2009), the provocative essay “The End of Theory” (Anderson, 2008) sparked intense debates by saying: “Who knows why people do what they do? The point is they do it, and we can track and measure it with unprecedented fidelity. With enough data, the numbers speak for themselves.” Yet, while the ability to capture large volumes of data brings along important scientific opportunities (Lazer et al., 2009; King, 2011), size by itself is not enough. Indeed, such claims were debunked by many critics (boyd and Crawford, 2012; Harford, 2014; Lazer et al., 2014; Giardullo, 2015; Hargittai, 2015), who emphasize that they ignore, among others, that size alone does not necessarily make the data better (boyd and Crawford, 2012) as “there are a lot of small data problems that occur in big data” which “don't disappear because you've got lots of the stuff. They get worse” (Harford, 2014).

Regardless of how large or varied social data are, there are also lingering questions about what can be learned from them about real-world phenomena (online or offline)—which have yet to be rigorously addressed (boyd and Crawford, 2012; Ruths and Pfeffer, 2014; Tufekci, 2014). Thus, given that these data are increasingly used to drive policies, to shape products and services, and for automated decision making, it is critical to gain a better understanding of the limitations around the use of various social datasets and of the efforts to address them (boyd and Crawford, 2012; O'Neil, 2016). Overlooking such limitations can lead to wrong or inappropriate results (boyd and Crawford, 2012; Kıcıman et al., 2014), which could be consequential particularly when used for policy or decision making.

At this point, a challenge for both academic researchers and applied data scientists using social data, is that there is not enough agreement on a vocabulary or taxonomy of biases, methodological issues, and pitfalls of this type of research. This review paper is intended for those who want to examine their own work, or that of others, through the lens of these issues.

### 1.2. Scope

While there is much research investigating social data and the various social and technical processes underlying its generation, most of the relevant studies do not position themselves within a framework that guides other researchers in systematically reasoning about possible issues in the social datasets and the methods they use. To this end, our goal is to gather evidence of a variety of different kinds of biases in social data, including their underlying social, technical and methodological underpinnings. While some of this evidence comes from studies that explicitly investigate a specific kind or source of bias; most of it comes from research that is leveraging social data with the goal of answering social science and social computing questions. That is, the evidence of bias and of broader implications about potential threats to the validity of social data research is often implicit in the findings of prior work, rather than a primary focus of it.

Through a synthesis of prior social data analyses, as well as literature borrowed from neighboring disciplines, we draw the connection between the patterns measured in various sources of online social data across diverse streams of literature and a variety of data biases, methodological pitfalls, and ethical challenges. We broadly categorize these biases and pitfalls as *manifestations* and *causes* of bias in order to better guide researchers who wish to systematically investigate bias-related risks as a result of their data and methods choice, as well as their implications for the stated research goals.

**Other systematic accounts of biases and dilemmas**. We recognize that some of the issues covered here are not unique to “social data," but instead relevant to data-driven research more broadly, and that aspects of these issues have been covered in other contexts as well (Friedman and Nissenbaum, 1996; Pannucci and Wilkins, 2010; Torralba and Efros, 2011). For instance, Pannucci and Wilkins (2010) study clinical trials that include a series of steps such planning, implementation, analysis/publication; and opportunities for bias at every step (similar to our idealized data pipeline section 2.3). Inspecting biases in data collections for object recognition, Torralba and Efros (2011) found that similar datasets merged together can be easily separated due to built-in biases: one can identify the dataset a specific data entry comes from. Broadly discussing bias in computer systems, Friedman and Nissenbaum (1996) characterize it according to its source such as societal, technical, or usage related. Theirs is, to the best of our knowledge, the first attempt to comprehensively characterize the issue of bias in computer systems, over 20 years ago.

**Because of far-reaching impact, biases in social data require renewed attention**. Social data has shaped entirely new research fields at the intersection of computer science and social sciences such as computational social science and social computing, fields that have also branched out into many neighboring application areas including crisis informatics, computational journalism, and digital health. As a cultural phenomenon, social media and other online social platforms have also provided a new expressive media landscape for billions of people, businesses, and organizations to communicate and connect, providing a window into social and behavioral phenomena at a large scale. Biases in social data and the algorithmic tools used to handle it can have, as a result, far-reaching impact. Further, while social datasets exhibit built-in biases due to how the datasets are created (González-Bailón et al., 2014a; Olteanu et al., 2014a), as is the case for other types of data, e.g., Torralba and Efros (2011), they also exhibit biases that are specific to social data, such as behavioral biases due to community norms (section 3.3).

**The term “bias.”** We also remark that “bias” is a broad concept that has been studied across many disciplines such as social science, cognitive psychology or law, and encompasses phenomena such as confirmation bias and other cognitive biases (Croskerry, 2002), as well as systemic, discriminatory outcomes (Friedman and Nissenbaum, 1996) or harms (Barocas et al., 2017). Oftentimes, however, it is difficult to draw clear boundaries between the more normative connotations and the statistical sense of the term—see Campolo et al. (2017) for a discussion on some of the competing meanings of the term “bias.” In this paper, we use the term mainly in its more statistical sense to refer to biases in social data and social data analyses (see our working definition of data bias in section 3.1).

### 1.3. Organization

**Figure 1:**
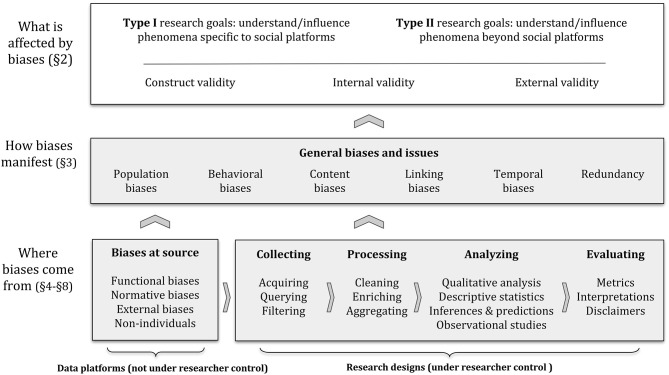
Depiction of the framework we use to describe biases and pitfalls when working with social data. The arrows indicate how components in our framework directly tend to affect others, indicating that reaching certain social data analysis goals (section 2.1) requires research to satisfy certain validity criteria (section 2.2), which can be compromised by biases and other issues with social data (section 3). These biases and issues may occur at the source of the data (section 4), or they may be introduced along the data analysis pipeline (sections 5–8). See section 2.3 for a more detailed description.

We begin our review by noting that whether a research method is adequate or not depends on the questions being asked and the data being used (section 2), and by covering a series of general biases and other issues in social data (section 3). While we note that research rarely happens in a linear fashion, we describe challenges along an idealized data pipeline, depicted in Figure 1. We first analyze problems at the data source (section 4) and introduced during data collection (section 5). Next, we describe issues related to data processing (section 6) and analysis (section 7), and issues that arise during the evaluation or interpretation of results (section 8). Finally, we discuss ethical issues of social data (section 9), before wrapping up with an brief overview of future directions (section 10).

## 2. Context and General Framework

Evaluating whether a dataset is biased or a methodology is adequate depends on the context in which research takes place, and fundamentally on the goals of the researcher(s). To better grasp how data and methodological issues might affect or shape research outcomes, we first describe the prototypical goals (section 2.1) and classes of validity threats (section 2.2) to social data research. We then overview our framework to describe biases and pitfalls in social data analysis along an illustrative vignette (section 2.3), showing how they can compromise research validity and goals.

### 2.1. Prototypical Goals of Social Data Analysis

Researchers and practitioners have explored the potential benefits of social data on a variety of domains and for many applications, of which we can broadly identify two classes of research goals:

- to understand or influence phenomena specific to social software platforms, often with the objective of improving them; or
- to understand or influence phenomena beyond social software platforms, seeking to answer questions from sociology, psychology, or other disciplines.

**Type I research** focuses on questions about social software platforms, including questions specific to a single platform or a family of related platforms, comparative analyses of platforms and of behaviors across platforms. This research typically applies methods from computer science fields such as data mining or human-computer interaction. This includes, for instance, research on maximizing the spread of “memes,” on making social software more engaging, and on improving the search engine or the recommendation system of a platform.

**Type II research** is about using data from social software platforms to address questions about phenomena that happens outside these platforms. This research may occur in emerging interdisciplinary domains, such as computational social science and digital humanities. Researchers addressing this type of problem may seek to use social data to answer questions and identify interventions relevant to media, governments, non-governmental organizations, and business, or to work on problems from domains such as health, economics, and education. Sometimes, the research question can be about the impact of social software platforms in these domains, e.g., to describe the influence of social media in a political election. In other cases, the goal may lie entirely outside social media itself, e.g., to use social data to help track the evolution of contagious diseases by analyzing symptoms reported online by social media users.

### 2.2. Validity of Social Data Research

To discuss validity threats to social data research, for illustrative purposes, let us assume that a researcher is analyzing social data to test a given hypothesis.^3^ A challenge, then, is an unaddressed issue within the design and execution of a study that may put the proof or disproof of the hypothesis into question. Social data research is often interdisciplinary, and as such, the vocabulary and taxonomies describing such challenges is varied (Howison et al., 2011; boyd and Crawford, 2012; Lazer et al., 2014). Without prejudice, we categorize them along the following classes of threats to the validity of research conclusions:

**Construct validity** or *Do our measurements over our data measure what we think they measure?* (Trochim and Donnelly, 2001; Quinn et al., 2010; Howison et al., 2011; Lazer, 2015). In general, a research hypothesis is stated as some assertion over a theoretical construct, or an assertion over the relationships between theoretical constructs. Construct validity asks whether a specific measurement actually measures the construct referred to in the hypothesis.

*Example:* If a hypothesis states that “self-esteem” increases with age, research tracking self-esteem over time from social media postings must ask whether its assessment of self-esteem from the postings actually measures self-esteem, or if instead it measures some other related or unrelated construct. In other words, we need to know whether the observed behaviors (such as words or phrases used in postings) are driven primarily by users' self-esteem vs. by community norms (section 4.2), system functionalities (section 4.1), or other reasons (section 3.3). Construct validity is specially important when the construct (self-esteem) is unobservable/latent and has to be operationalized via some observed attributes (words or phrases used).

**Internal validity** or *Does our analysis correctly lead from the measurements to the conclusions of the study?* Internal validity focuses on the analysis and assumptions about the data (Howison et al., 2011). This survey covers subtle errors of this kind, such as biases that can be introduced through data cleaning procedures (section 6), the use of machine learned classifiers, mistaken assumptions about data distributions, and other inadvertent biases introduced through common analyses of social media (section 7).

*Example:* An analysis of whether “self-esteem” increases with age may not be internally valid if text filtering operations accidentally remove terms expressing confidence (section 5.3); or if machine learned classifiers were inadvertently trained to recognize self-esteem only in younger people (section 7). Of course, while we do not dwell on them, researchers should also be aware of more blatant logical errors—e.g., comparing the self-esteem of today's younger population to the self-esteem of today's older population would not actually prove that self-esteem increases with age (section 3.6).

**External validity** or *To what extent can research findings be generalized to other situations?* Checking external validity requires to focus on ways in which the experiment and the analysis may not represent the broader population or situation (Trochim and Donnelly, 2001). For example, effects observed on a social platform may manifest differently on other platforms due to different functionalities, communities, or cultural norms (Wijnhoven and Bloemen, 2014; Malik and Pfeffer, 2016). The concept of external validity includes what is sometimes called *ecological validity*, which captures to what extent an artificial situation (constrained social media platform) properly reflects a broader real-world phenomenon (Ruths and Pfeffer, 2014). It also includes *temporal validity*, which captures to what extent constructs change over time (Howison et al., 2011) and may invalidate previous conclusions about societal and/or platform phenomena; e.g., see the case of Google Flu Trends (Lazer et al., 2014).

*Example:* Even after we conclude a successful study of “self-esteem” in a longitudinal social media dataset collected from a given social media platform (section 4), its findings may not generalize to a broader setting as people who chose that particular platform may not be representative of the broader population (section 3.2); or perhaps their behaviors online are not representative of their behaviors in other settings (section 3.3).

Each of these validity criteria is complex to define and evaluate, being general to many types of research beyond social data analyses; the interested reader can consult (Trochim and Donnelly, 2001; Quinn et al., 2010; Howison et al., 2011; Lazer, 2015). Specific challenges are determined by the objectives and the research questions one is trying to answer. For instance, a study seeking to improve the ordering of photos shown to users on one photo sharing site may not need to be valid for other photo sharing sites (external validity). In contrast, a study concerned with how public health issues in a country are reflected on social media sites may aspire to ensure that results are independent of the websites selected for the study.

### 2.3. A Framework to Describe Biases and Pitfalls in Social Data

As depicted in Figure 1, social data analysis starts with certain *goals* (section 2.1), such as understanding or influencing phenomena specific to social platforms (Type I) and/or phenomena beyond social platforms (Type II). These *goals* require that research satisfies certain *validity* criteria, described earlier (section 2.2). These criteria, in turn, can be compromised by a series of *general biases and issues* (section 3). These challenges may depend on the characteristics of each *data platform* (section 4)—which are often not under the control of the researcher—and on the *research designs* choices made along a data processing pipeline (from sections 5 to 8)–which are often under the researcher control.

In this paper, for each type of bias we highlight, we include a *definition* (provided at the general level), the *implications* of the issue in terms of how it affects research validity and goals, and a list of *common issues* that we have identified based on prior work. We believe this organization facilitates the adoption by researchers and practitioners, as for practical reasons they can be assumed to know details about their own research design choices, even if other elements in the framework may be less explicitly considered.

**Example/vignette:** Let us consider another brief hypothetical example. Suppose we are interested in determining the prevalence of dyslexia in different regions or states of a country, and we decide to use observations of writing errors in social media postings to try answer this question.^4^ First, we determine that the research goal is of *type II*, as it seeks to answer a question that is external to social data platforms. Second, we consider different aspects of research validity. With respect to *construct validity* we observe that the literature on dyslexia indicates that in some cases this disorder is often noticeable in the way people write, but not always; hence we note the type of dyslexia we will be able to capture. With respect to *internal validity* we need to determine and describe the extent to which our method for content analysis will reflect this type of dyslexia. With respect to *external validity* we need to note the factors that may affect the generalization of our results.

Third, we consider each potential data bias; for illustrative purposes we mention *population biases* (section 3.2), *content biases* (section 3.4), *functional biases* (section 4.1), and *normative biases* (section 4.2). With respect to population biases, we need to understand to what extent the demographic characteristics of the population we sample from social media reflect those of the country's population, e.g., whether users are skewed toward the younger. Regarding content biases, we note the need to consider the effects of the context in which people write on social media and the attention they put in writing correctly. For functional biases, we need to note whether the platforms we study include functionalities that may affect our results, such as a spell-checker, and whether those functionalities are enabled by default. Regarding normative biases, we need to account for how users are expected to write in a site, which would be different, e.g., in a job search vs. an anonymous discussion site.

Fourth, we map these issues to choices in our research design. For example, data *querying* (section 5) should not be based on keywords that might be misspelled. Data *cleaning* (section 6) should not involve text normalization operations that may affect the writing patterns we want to capture. During the data *analysis* (section 7) we need to correctly separate different factors that may lead to a writing mistake, seeking ways to isolate dyslexia (e.g., by using a sample of texts written by people with dyslexia). Finally, the *interpretation* of our results (section 8) needs to be consistent with the elements that may affect our research design.

## 3. General Biases and Issues

General challenges for research using social data include population biases (section 3.2), behavioral biases (section 3.3), content biases (section 3.4), linking biases (section 3.5), temporal variations (section 3.6), and redundancy (section 3.7). To situate these issues within the wider concept of data quality, we begin by briefly overviewing known data quality issues.

### 3.1. Data Quality

Data quality is a multifaceted concept encompassing an open-ended list of desirable attributes such as completeness, correctness, and timeliness; and undesirable attributes such as sparsity and noise, among others. The impact of these attributes on specific issues varies with the analysis task. In general, data quality bounds the questions that can be answered using a dataset. When researchers gather social datasets from platforms outside their control, they often have little leverage to control data quality.

Two well-known shortcomings in social data quality are sparsity and noise:

–* Sparsity*. Many measures follow a power-law or heavy-tailed distribution,^5^ which makes them easier to analyze on the “head” (in relation to frequent elements or phenomena), but difficult on “tail” (such as rare elements or phenomena) (Baeza-Yates, 2013). This can be exacerbated by platform functionality design (e.g., limiting the length of users' posts) (Saif et al., 2012), and may affect, for instance, data retrieval tasks (Naveed et al., 2011).^6^

–* Noise*. Noise refers to content that is incomplete, corrupted, contains typos/errors, or content that is not reliable or credible (Naveed et al., 2011; boyd and Crawford, 2012). The distinction between what is “noise” and what is “signal” is often unclear, subtly depending on the research question (Salganik, 2017); the problem rather being finding the *right data* (Baeza-Yates, 2013)–simply adding more data may increase the level of noise and reduce the quality and reliability of results.

Another important data quality issue, and the main focus of this paper, is *data bias*.

**Definition (Data bias)**. A systematic distortion in the sampled data that compromises its representativeness.

Most research on social data uses a fraction of all available data (a “sample”) to learn something about a larger population. Sampling is so prevalent that we rarely question it. Thus, in many of these scenarios, the samples should be *representative* of a larger population of interest, defined with respect to criteria such as *demographic characteristics* (e.g., women over 55 years old) or *behavior* (e.g., people playing online games). Samples should also represent well the content being produced by different groups.

Determining representativeness is complicated when the available data does not fully capture the relevant properties of either the sampled users or the larger population. Considering the classification in section 2.1, sample representativeness affects research questions of type I (internal to a platform) that may need to focus on certain subgroups of users. Yet, identifying such groups is not trivial, as the available data may not capture all relevant properties of users. Research questions of type II (about external human phenomena) are further complicated. Often, they come along with a definition of a target population of interest (Ruths and Pfeffer, 2014) such as estimating the political preferences of young female voters or of citizens with a college degree.

Furthermore, there may also be multiple ways to express representativesness objectives (sections 3.2–3.6), including with respect to some target population, to some notion of relevant content, or to some reference behavior. However, obtaining a uniform random sampling may be difficult or impossible when acquiring social data (see section 5). Data quality will depend on the interplay of sample sizes and various sample biases—for a detailed and formal treatment of these issues, see Meng (2018). Data biases are often evaluated by comparing a data sample with reference samples drawn from different sources or contexts. Thus, data bias is rather a *relative* concept (Mowshowitz and Kawaguchi, 2005). For instance, when we speak of “content production biases” (section 3.4), we often mean that the content in two social datasets may systematically differ, even if the users writing those contents overlap.

### 3.2. Population Biases

**Definition (Population biases)**. Systematic distortions in demographics or other user characteristics between a population of users represented in a dataset or on a platform and some target population.

The relationship between a studied population (e.g., adults on Twitter declaring to live in the UK) and a target population (e.g., all adults living in the UK) is often unknown. In general, both early surveys from the Pew Research Center and academic studies (Hargittai, 2007; Mislove et al., 2011) show that the demographic composition of major social platforms differs both with respect to each other, and with respect to the offline or Internet population (see e.g., Duggan, 2015 for the US).^7^ In other words, it shows that individuals do not randomly self-select when using social media platforms (Hargittai, 2015), with demographic attributes such as age, gender, race, socioeconomic status, and Internet literacy correlating with how likely someone is to use a social platform.

**Implications**. Population biases may affect the representativeness of a data sample and, as a result, may compromise the ecological/external validity of research. They are particularly problematic for research of type II (section 2.1), where conclusions about society are sought from social data, such as studies of public opinion. They can also impact the performance of algorithms that make inferences about users (Johnson et al., 2017), further compromising the internal validity of both type I and type II research.

**Common issues**. Three common manifestations of population bias are the following:

–* Different user demographics tend to be drawn to different social platforms*. Prior surveys and studies on the use of social platforms show differences in gender representation across platforms (Anderson, 2015), as well as race, ethnicity, and parental educational background (Hargittai, 2007). For instance, Mislove et al. (2011) found that Twitter users significantly over-represent men and urban populations, while women tend to be over-represented on Pinterest (Ottoni et al., 2013).

–* Different user demographics use platform mechanisms differently*. Prior work showed that people with different demographic, geographic, or personality traits sometimes use the same platform mechanisms for different purposes or in different ways. For instance, users of different countries tend to use Twitter differently—Germans tend to use hashtags more often (suggesting a focus on information sharing), while Koreans tend to reply more often to each other (suggesting a focus on conversations) (Hong et al., 2011). Another example is a question-answering site where the culture encourages hostile corrections, driving many users to remain “unregistered and passive.^8^” Thus, studies assuming a certain usage may misrepresent certain groups of users.

–* Proxies for user traits or demographic criteria vary in reliability*. Most users do not self-label along known demographic axes. For example, a study interested in the opinion of young college graduates about a new law may rely on a proxy population: those reporting on a social platform to be alumni of a given set of universities. This choice can end up being an important source of bias (Ruths and Pfeffer, 2014). In the context of predicting users' political orientations, researchers have shown that the choice of the proxy population drastically influences the performance of various prediction models (Cohen and Ruths, 2013).

### 3.3. Behavioral Biases

**Definition (Behavioral biases)**. Systematic distortions in user behavior across platforms or contexts, or across users represented in different datasets.

Behavioral biases appear across a wide range of user actions, including how they connect and interact with each other, how they seek information, and how they create and appraise content.^9^ For instance, studies looking at similarities and differences among social platforms found differences with respect to user personalities (Hughes et al., 2012), news spreading (Lerman and Ghosh, 2010), or content sharing (Ottoni et al., 2014).

**Implications**. Behavioral biases affect the ecological/external validity of research, as they may condition the results of a study on the chosen platform or context. They are not entirely dependent on population biases, and when (explicit or implicit) assumptions are made about the users' behavioral patterns, they can affect both type I and II research (section 2.1) that, e.g., looks into users' needs or interests, among others.

**Common issues**. We separately discuss behavioral biases affecting the generation of content by users (section 3.4) and those affecting linking patterns between users (section 3.5). Three other common classes of behavioral biases involve interactions among users, interactions between users and content, and the biases that cause users to be included or excluded from a study population.

–* Interaction biases affect how users interact with each other*. Differences in how people communicate are influenced by shared relationships, norms, and platform affordances. while others indicate that how users interact depends on the type of relation they share (Burke et al., 2013) and on shared characteristics (i.e., homophily) (McPherson et al., 2001).

–* Content consumption biases affect how users find and interact with content, due to differences in their interests, expertise, and information needs*. Studying web search behavior, Silvestri (2010) found that it varies across semantic domains, and Aula et al. (2010) that it changes as the task at hand becomes more difficult. By observing the interplay between what people search and share about health on social media, De Choudhury et al. (2014) found information seeking and sharing practices to vary with the characteristics of each medical condition, such as its severity.

Further, people's consumption behavior is correlated with their demographic attributes and other personal characteristics: Kosinski et al. (2013) links “likes” on Facebook with personal traits, while web page views vary across demographics, e.g., age, gender, race, income (Goel et al., 2012). Since users tend to consume more content from like-minded people, such consumption biases were linked to the creation of filter bubbles (Nikolov et al., 2015).

–* Self-selection and response bias may occur due to behavioral biases*. Studies relying on self-reports may be biased due to *what* users choose to report or share about, *when* they report it, and *how* they choose to do it. This can happen either because their activities are not visible (e.g., a dataset may not include people who only read content without writing), or due to self-censorship (e.g., not sharing or deleting a post) as a result of factors such as online harassment, privacy concerns, or some sort of social repercussion (Wang et al., 2011; Das and Kramer, 2013; Matias et al., 2015).

Apart from “missing” reports, inaccurate self-reports can also bias social datasets—often termed as *response bias*.^10^ Zhang et al. (2013) found that about 75% of Foursquare check-ins do not match users' real mobility, being influenced by Foursquare's competitive game-like mechanisms (Wang et al., 2016a). Further, users are more likely to talk about extreme or positive experiences than common or negative experiences (Kícíman, 2012; Guerra et al., 2014). Response bias may also affect the use of various platforms mechanisms; Tasse et al. (2017) show that while some users do not know they are geotagging their social media posts, many users consciously use geotagging to e.g., show off were they have been.

When overlooked, such reporting biases can also lead to discrimination (Crawford, 2013; Barocas, 2014). For example, data-driven public policies may only succeed in economically advantaged, urban, and data-rich areas (Hecht and Stephens, 2014; Shelton et al., 2014), if efforts are not made to improve data collection elsewhere (see the “digital divide” on section 9.4).

### 3.4. Content Production Biases

**Definition (Content production biases)**. Behavioral biases that are expressed as lexical, syntactic, semantic, and structural differences in the content generated by users.

**Implications**. The same as for behavioral biases: content production biases affect ecological/external validity of both type I and II research. Further, these biases raise additional concerns as they affect several popular tasks, such as user classification, trending topics detection, language identification, or content filtering (Cohen and Ruths, 2013; Olteanu et al., 2014a; Blodgett et al., 2016; Nguyen et al., 2016), and may also impact users' exposure to a variety of information types (Nikolov et al., 2015).

**Common issues**. Variations in user generated content, particularly text, are well-documented across and within demographic groups.

–* The use of language(s) varies across and within countries and populations*. By mapping the use of languages across countries, Mocanu et al. (2013) observed seasonal variations in the linguistic composition of each country, as well as between geographical areas at different granularity scales, even at the level of city neighborhoods. Such variations were also observed across racial or ethnic groups (Blodgett et al., 2016).

–* Contextual factors impact how users talk*. The use of language is shaped by the relations among people; Further, Schwartz et al. (2015) show that the temporal orientation of messages (emphasizing the past, present, or future) may be swayed by factors like openness to new experiences, number of friends, satisfaction with life, or depression.

–* Content from popular or “expert” users differs from regular users' content*. For instance, Bhattacharya et al. (2014) found that on Twitter “expert” users tend to create content mainly on their topic of expertise, while

–* Different populations have different propensities to talk about certain topics*. For instance, by selecting political tweets during the 2012 US election, Diaz et al. (2016) noticed a user population biased toward Washington, DC; while Olteanu et al. (2016) found African-Americans more likely to use the #BlackLivesMatter Twitter hashtag (about a large movement on racial equality in the US).

### 3.5. Linking Biases

**Definition (Linking biases)**. Behavioral biases that are expressed as differences in the attributes of networks obtained from user connections, interactions or activity.

**Implications**. The social networks (re)constructed from observed patterns in social datasets may be fundamentally different from the underlying (offline) networks (Schoenebeck, 2013a), posing threats to the ecological/external validity. This is particularly problematic for type II research and, in cases where user interaction or linking patterns vary with time or context, it can also affect type I research. Linking biases impact the study of, e.g., social networks structure and evolution, social influence, and information diffusion phenomena (Wilson et al., 2009; Cha et al., 2010; Bakshy et al., 2012). On social platforms, they may also result in systematically biased perceptions about users or content (Lerman et al., 2016).

**Common issues**. Types of manifestations for linking biased include:

–* Network attributes affect users' behavior and perceptions, and vice versa*. while Dong et al. (2016) found age-specific social network distances (“degrees of separation”), with younger people being better connected than older generations. Further, *homophily*—the tendency of similar people to interact and connect (McPherson et al., 2001)—can systematically bias the perceptions of networked users, resulting in under- or over-estimations of the prevalence of user attributes within a population (Lerman et al., 2016).

In social datasets, linking biases can be further exacerbated by how data is collected and sampled, and by how links are defined, impacting the observed properties of a variety of network-based user attributes, such as their centrality within a social network (Choudhury et al., 2010; González-Bailón et al., 2014b) (see also section 5).

–* Behavior-based and connection-based social links are different*. Different graph construction methods can lead to different structural graph properties in the various kinds of networks that can be constructed from social data (Cha et al., 2010). Exploring the differences between social networks based on explicit vs. implicit links among users, Wilson et al. (2009) also showed that the network constructed based on explicit links was significantly denser than the one based on user interactions.

–* Online social networks formation also depends on factors external to the social platforms*. Geography has been linked to the properties of online social networks (Poblete et al., 2011; Scellato et al., 2011), with the likelihood of a social link decreasing with the distance among users, with consequences for information diffusion (Volkovich et al., 2012). Further, the type and the dynamics of offline relationships influence users propensity to create social ties and interact online (Subrahmanyam et al., 2008; Gilbert and Karahalios, 2009).

### 3.6. Temporal Biases

**Definition (Temporal Biases)**. Systematic distortions across user populations or behaviors over time.

Data collected at different points in time may differ along diverse criteria, including who is using the system, how the system is used, and in the platform affordances. Further, these differences may exhibit a variety of patterns over time, including with respect to granularity and periodicity.

**Implications**. Temporal biases affect both the internal and ecological/external validity of social data research. They are problematic for both type I and II research (section 2.1), as they may affect the generalizability of observations over time (e.g., what factors vary and how they confound with the current patterns in the data). If a platform and/or the offline context are not stable, it may be impractical to disentangle the effects due to a specific variable of interest from variations in other possible confounding factors.

**Common issues**. How one aggregates and truncates datasets along the temporal axes impacts what type of patterns are observed and what research questions can be answered.

–* Populations, behaviors, and systems change over time*. Studies on both Facebook (Lampe et al., 2008) and Twitter (Liu et al., 2014) have found evidence of such variations. Even the user demographic composition and participation on a specific topic (Guerra et al., 2014; Diaz et al., 2016) or their interaction patterns (Viswanath et al., 2009) are often non-stationary. There are often complex interplays between behavioral trends on a platform (e.g., in the use of language) and the online communities' makeup and users' lifecycles (Danescu-Niculescu-Mizil et al., 2013), which means sometimes change happens at multiple levels. For instance, there are variations on when and for how long users focus on certain topics that may be triggered by current trends, seasonality or periodicity in activities, or even noise (Radinsky et al., 2012). Such trends can emerge organically or be engineered through platform efforts (e.g., marketing campaigns and new features).

–* Seasonal and periodic phenomena*. These can trigger systematic variations in usage patterns (Radinsky et al., 2012; Grinberg et al., 2013). When analyzing geo-located tweets, Kıcıman et al. (2014) found that different temporal contexts (day vs. night, weekday vs. weekend) changed the shapes of inferred neighborhood boundaries. while Golder and Macy (2011) observed links between the sentiment of tweets and cycles of sleep and seasonality.

–* Sudden-onset phenomena affect populations, behaviors, and platforms*. Examples include suddenly emerging data patterns (e.g., a spike or drop in activity) due to external events (e.g., an earthquake or accident) or platform changes. while real-world events like crisis situations may result in short-lived activity peaks (Crawford and Finn, 2014).

–* The time granularity can be too fine-grained to observe long-term phenomena*. Examples include phenomena maintaining fairly constant patterns or evolving over long periods (Richardson, 2008; Crawford and Finn, 2014). For instance, while social datasets related to real-world events are often defined around activity peaks, distinct events may have different temporal fingerprints that such datasets may miss (e.g., disasters may have longer-term effects than sport events). The temporal fingerprints of protracted situations like wars may also be characterized by multiple peaks. Others observed that long-term search logs (vs. short-term, within-session search information) provide richer insights into the evolution of users' interests, needs, or how their experiences unfold (Richardson, 2008; Fourney et al., 2015).

–* The time granularity can be too coarse-grained to observe short-lived phenomena*. This is important when tracking short-lived effects of some experience, or smaller phenomena at the granularity of, e.g., hours of minutes. showing that how users' timelines are aligned and truncated may influence what patterns they capture. Further, some of the patterns and correlations observed in social data may be evolving or may be only short-lived (Starnini et al., 2016).

–* Datasets decay and lose utility over time*. Social data decays with time as users delete their content and accounts (Liu et al., 2014; Gillespie, 2015), and platforms APIs' terms of service prevent sharing of datasets as they are collected. This often makes it impractical to fully reconstruct datasets over time, leaving important holes (“Swiss Cheese” decay) (Bagdouri and Oard, 2015). while Almuhimedi et al. (2013) that about 2.4% of tweets posted during 1 week in 2013 by about 300 million users, were later deleted (with about half of users deleting at least one tweet from that period).

There are several mechanisms rendering a message unavailable later on (Liu et al., 2014): it was explicitly deleted by the user; the user switched their account to “protected” or private; the user's account was suspended by the platform; or the user deactivated their entire account. Yet, often, it may be unclear why certain posts were removed and may be hard to gauge their impact on analysis results.

### 3.7. Redundancy

**Definition (Redundancy)**. Single data items that appear in the data in multiple copies, which can be identical (duplicates), or almost identical (near duplicates).

**Implications**. Redundancy, when unaccounted for, may affect both the internal and ecological/external validity of research, in both type I and type II research (section 2.1). It may negatively impact the utility of tools (Radlinski et al., 2011), and distort the quantification of phenomena in the data.

**Common issues**. Lexical (e.g., duplicates, re-tweets, re-shared content) and semantic (e.g., near-duplicates or same meaning, but written differently) redundancy often accounts for a significant-fraction of content (Baeza-Yates, 2013), and may occur both within and across social datasets.

Other sources of content redundancy often include non-human accounts (section 4.4) such as the same entity posting from multiple accounts or platforms (e.g., spam), multiple users posting from the same account (e.g., organization accounts), or multiple entities posting or re-posting the same content (e.g., posting quotes, memes, or other types of content). This can sometimes distort results, yet, redundancy can be a signal by itself, for instance, reposting may be a signal of importance.

## 4. Issues at the Data Source or Origin

The behaviors we observe in online social platforms are partially determined by platform capabilities, which are engineered toward certain goals (Van Dijck, 2013a; Tufekci, 2014; Gillespie, 2015; Salganik, 2017). We first overview biases due to platform design and affordances (section 4.1) and due to behavioral norms that exist or emerge on each platform (section 4.2). Then, we examine factors external to social platforms, but which may influence user behavior (section 4.3). Finally, we briefly discuss the presence of non-individual accounts (section 4.4).

### 4.1. Functional Biases

**Definition (Functional biases)**. Biases that are a result of platform-specific mechanisms or affordances, that is, the possible actions within each system or environment.

Platform affordances are often driven by business considerations and interests (Van Dijck, 2013a; Salganik, 2017), and influenced by the politics, assumptions, and interests of those designing and building these platforms (Van Dijck, 2013a; West et al., 2019). Platform affordances and features are sometimes purposefully introduced to “nudge” users toward certain behaviors (Thaler and Sunstein, 2008). Each platform uses unique, proprietary, and often undocumented platform-specific algorithms to organize and promote content (or users), affecting user engagement and behavior. Ideally, research should use social data samples from different platforms, but due to limits in the availability of data, much research is concentrated on data from a few platforms, most notably Twitter. Its usage as a sort of “model organism” by social media research has been criticized (Tufekci, 2014).

**Implications**. Functional biases make conclusions from research studies difficult to generalize or transfer, as each platform exhibits its own structural differences (Tufekci, 2014), which can lead to platform-specific phenomena (Ruths and Pfeffer, 2014) that are often overlooked. The fact that most research is done using data from a handful of platforms makes this issue even more severe. Functional biases are problematic for type II research (section 2.1), affecting the external/ecological validity of social data research; and, when e.g., they change over time, they can also affect type I research. Their influence on behavior and adoption patterns, however, is often subtle and hard to disentangle from other factors.

**Common issues**. The functional peculiarities of social platforms may introduce population (section 3.2) and behavioral biases (section 3.3) by influencing which user demographics are drawn to each platform and the kind of actions they are more likely to perform (Tufekci, 2014; Salganik, 2017). Manifestations of functional biases include:

–* Platform-specific design and features shape user behavior*. Through randomized experiments (A/B tests) and longitudinal observations, one can observe how new features and feature changes impact usage patterns on social platforms. For example, Facebook observed that decreasing the size of the input area for writing a reply to a posting resulted in users sending shorter replies, faster, and more frequently.^11^ On Twitter, Pavalanathan and Eisenstein (2016) found that the introduction of emojis lead to a decrease in the usage of emoticons.

The effects of platform design on user behavior can also be observed through comparative studies. Both platforms allow book reviewing and rating, yet differ in the content of the reviews, the ratings, and how reviews get promoted. Users seem aware of such differences across platforms, highlighting key features in their adoption of a platform such as interface aesthetics, voting functionality, community size, as well as the diversity, recency and the quality of the available content (Newell et al., 2016b).

–* Algorithms used for organizing and ranking content influence user behavior*. User engagement with content and other users is influenced by algorithms that determine *what* information is shown, *when* is it shown, and *how* is it shown. This has been dubbed “algorithmic confounding” (Salganik, 2017). A illustrative example is a ranked list of content (e.g., search results) that “buries” content found in the lower positions, due to click and sharing bias or users perception of higher ranked content as being more trustworthy (Hargittai et al., 2010). This may provide an advantage to, e.g, certain ideological or opinion groups (Liao et al., 2016). Personalized rankings further complicate these issues. Hannak et al. (2013) observe that, on average, about 12% of Google search results exhibit differences due to personalization. This has important societal implications as it can lead to less diverse exposure to content, or to being less exposed to content that challenges one's views (Resnick et al., 2013).

–* Content presentation influences user behavior*. How different aspects of a data item are organized and emphasized, or how various data items are represented, also impact how users interact with and interpret them across platforms. For instance, Miller et al. (2016) show that variations in how emojis are displayed across smartphones can lead to confusion among users, as different renderings of the same concept are so different that they might be interpreted as having different meanings and emotional valence.

### 4.2. Normative Biases

**Definition (Normative biases)**. Biases that are a result of written or unwritten norms and expectations of acceptable patterns of behavior on a given online platform or medium.

Platforms are characterized by their behavioral norms, usually under the form of expectations about what constitutes acceptable use. These norms are shaped by factors including the specific value proposition of each platform, and the composition of their user base (boyd and Ellison, 2007; Ruths and Pfeffer, 2014; Newell et al., 2016b). For instance, Sukumaran et al. (2011) shows how news websites' users conform to informal standards set by others of when writing comments like length or number of covered aspects.

**Implications**. As with functional biases, normative biases affect the ecological/external validity of research, and are problematic for type II research (section 2.1), since research results may depend on the particular norms of each platform. They can also distort user behavior and tend to vary with context, time, or across sub-communities, also affecting type I research. Overlooking the impact of norms can impact any social data analysis studying or making assumptions about user behavior (Tufekci, 2014).

**Common issues**. There is a complex interplay between norms, platforms, and behaviors:

–* Norms are shaped by the attitudes and behaviors of online communities, which may be context-dependent*. Other elements, such as design choices, explicit terms of use, moderation policies, and moderator activities, also affect norms. Users may exhibit different behavioral patterns on different platforms (Skeels and Grudin, 2009): e.g., they may find acceptable to share family photos on Facebook, but not on LinkedIn (Van Dijck, 2013b).^12^ Norms are also sensitive to context, as the meaning of the same action or mechanism may change under different circumstances (Freelon, 2014). They may also change over time due to e.g., demographic shifts (McLaughlin and Vitak, 2012; Ruths and Pfeffer, 2014).

–* The awareness of being observed by others impacts user behavior*. People try to influence the opinion that others form about them by controlling their own behavior (Goffman, 1959), depending on who they are interacting with, and the place and the context of the interactions. In online scenarios, users often navigate how to appropriately present themselves depending on their target or construed audience (Marwick and Boyd, 2011). Besides self-presentation, privacy concerns may also affect what users do or share online (Acquisti and Gross, 2006).

Online, the observers may include platform administrators, platform users, or researchers, in what we dub “online” Hawthorne effect.^13^ For instance, users are more likely to share unpopular opinions or to make sensitive or personal disclosures in private or anonymous spaces, than they are to do so in public ones (Bernstein et al., 2011; Schoenebeck, 2013b; Shelton et al., 2015). It has been observed that users who disclose their real name are less likely to post about sensitive topics, compared to users who use a pseudonymous (Peddinti et al., 2014). Users were also found to be more likely to “check-in” at public locations (e.g., restaurants) than at private ones (e.g., a doctor's office) (Lindqvist et al., 2011).

–* Social conformity and “herding” happen in social platforms, and such behavioral traits shape user behavior*. For instance, prior ratings and reviews introduce significant bias in individual rating behavior and writing style, creating a *herding effect* (Muchnik et al., 2013; Michael and Otterbacher, 2014).

### 4.3. External Sources of Bias

**Definition (External biases)**. Biases resulting from factors outside the social platform, including considerations of socioeconomic status, ideological/religious/political leaning, education, personality, culture, social pressure, privacy concerns, and external events.

Social platforms are open to the influence of a variety of external factors that may affect both the demographic composition and the behavior of their user populations.

**Implications**. External biases are a broad category that may affect construct, internal, and external validity of research, and be problematic for both type I and type II research (section 2.1). In general, external factors may impact various quality dimensions of social datasets, including coverage and representativeness, yet they can also be subtle and easy to overlook, and affect the reliability of observations drawn from these datasets (Silvestri, 2010; Kícíman, 2012; Olteanu et al., 2015).

**Common issues**. We cover several types of extraneous factors including social and cultural context, external events, semantic domains and sources.

–* Cultural elements and social contexts are reflected in social datasets*. The demographic makeup of a platform's users has an effect on the languages, topics, and viewpoints that are observed (Preoţiuc-Pietro et al., 2015) (see also population biases, section 3.2). The effect of a particular culture is typically demonstrated through transversal studies comparing a platform's usage across countries. However, as the social context changes in each country, these effects may vary over time.

For instance, a user's country of origin was shown to be a key factor in predicting their questioning and answering behavior (Yang et al., 2011), and may explain biases observed in geo-spatial social datasets such as OpenStreetMap (Quattrone et al., 2015). Hence, it is important for guiding the design of cross-cultural tools (Hong et al., 2011; Yang et al., 2011), and for understanding socio-economic phenomena (Garcia-Gavilanes et al., 2013). In addition to cultural idiosyncrasies, the broader social context of users (including socio-economic or demographic factors) also plays a role in users' behaviors and interactions. For instance, the way in which users are perceived affects their interaction patterns (e.g., the volume of shared content or of followees), as well as their visibility on a platform (e.g., how often they are followed, added to lists, or retweeted) (Nilizadeh et al., 2016; Terrell et al., 2016).

–* As other media, social media contains misinformation and disinformation*. Misinformation is false information unintentionally spread, while disinformation is false information that is deliberately spread (Stahl, 2006). Users post misinformation due to errors of judgment, while disinformation is often posted purposefully (e.g., for political gain). Disinformation can take forms beyond the production of hoaxes or “fake news” (Lazer et al., 2018); and include other types of manipulation of social systems, such as Google bombs to associate a keyword to a URL by repeatedly searching for the keyword and clicking on the URL, or review spam (Jindal and Liu, 2008) to manipulate the reputation of a product/service.^14^

Both types of false information can distort social data, sometimes in subtle ways. Of the two, one could argue that disinformation is harder to deal with, since it occurs in an adversarial setting and the adversaries can engage in an escalation of countermeasures to avoid detection. While past studies show that such content is rarely shared, its' consumption is concentrated within certain groups like older users (Grinberg et al., 2019; Guess et al., 2019). Approaches to mitigate the effects of misinformation and disinformation exist, including graph-based (Ratkiewicz et al., 2011), and text-based (Castillo et al., 2013) methods.

–* Contents on different topics are treated differently*. This is notable with respect to sharing, attention, and interaction patterns. In addition, due to both automated mechanisms and human curation, social media also exhibits common forms of bias present in traditional news media (Lin et al., 2011; Saez-Trumper et al., 2013), including gatekeeping (preference for certain topics), coverage (disparity in attention), and statement bias (differences in how an issue is presented) (D'Alessio and Allen, 2000).

–* High-impact events, whether anticipated or not, are reflected on social media*. Just as in news media, high-impact sudden-onset events (e.g., disasters) and seasonal cultural phenomena (e.g., Ramadan or Christmas) tend to be covered prominently on social media. Their prominence affects not only how likely users are to mention them, but also what they say (Olteanu et al., 2017b); just as the characteristics of crisis events leave a distinctive “print” on social media with respect to time and duration, including variations in the kind of information being posted and by whom (Saleem et al., 2014; Olteanu et al., 2015).

### 4.4. Non-individual Accounts

**Definition (Non-individual agents)**. Interactions on social platforms that are produced by organizations or automated agents.

**Implications**. Researchers often assume each account is an individual; when this does not hold, internal and external validity can be affected in both type I and type II research (section 2.1). For instance, studies using these datasets to make inferences about the prevalence of different opinions among the public may be particularly affected.

**Common issues**. There are two common types of non-individual accounts:

–* Organizational accounts*. Researchers have noted that “studies of human behavior on social media can be contaminated by the presence of accounts belonging to organizations” (McCorriston et al., 2015). Note that it is common practice for organizations (such as NGOs, government, businesses, and media) to have an active presence on social media. For instance, in a study of the #BlackLivesMatter movement, about 5% of the Twitter accounts that included the #BlackLivesMatter hashtag in their tweets were organizations (Olteanu et al., 2016). Furthermore, organizational accounts may produce more content than regular accounts (over 60% of the overall content in one study Olteanu et al., 2015).

–* Bots*. Bots and spamming accounts are increasingly prevalent (Abokhodair et al., 2015; Ferrara et al., 2016). Such accounts use tricks such as hijacking “trending” hashtags of keywords to gain visibility (Thomas et al., 2011), and can (purposefully or accidentaly) distort the statistics of datasets collected from social platforms (Morstatter et al., 2016;Pfeffer et al., 2018).

However, not all automated accounts are malicious. Some of them are used to post important updates about weather or other topics, such as emergency alerts. Others are designed by third party researchers to understand and audit system behavior (Datta et al., 2015). Broadly, the challenge is how to effectively separate them from accounts operated by individual users (boyd and Crawford, 2012; Ruths and Pfeffer, 2014). In fact, some users mix manual postings with automated ones, resulting in accounts that blend human with bot behavior—dubbed *cyborgs* (Chu et al., 2012). These cases are particularly difficult to detect and account for in an analysis. Further, simply identifying and removing bots from the analysis may be insufficient, as the behavior of such accounts (e.g., what content they post or who they “befriend”) influences the behavior of human accounts as well (Wagner et al., 2012).

## 5. Issues Introduced while Collecting Data

**Definition (Data collection biases)**. Biases introduced due to the selection of data sources, or by the way in which data from these sources are acquired and prepared.

Social datasets are strongly affected by their origin due to platform-specific phenomena: users of different platforms may have different demographics (population biases, section 3.2), and may behave differently (section 3.3) due to functional (section 4.1) and normative biases (section 4.2). This section examines issues resulting from data acquisition (section 5.1), of querying data APIs (section 5.2), and of (post-)filtering (section 5.3).

**Implications**. The ways in which the choicei of certain data sources affects the observations one makes, and thus the research results, can be described as *source selection bias*, affecting the external/ecological validity of type II research (section 2.1). Beyond source selection bias, several aspects related to how data samples are collected from these sources have been questioned, including their representativeness and completeness (González-Bailón et al., 2014b; Hovy et al., 2014), which is problematic for both type I and type II research.

### 5.1. Data Acquisition

Acquisition of social data is often regulated by social platforms, and hinges on the data they capture and make available, on the limits they may set to access, and on the way in which access is provided.

**Common issues**. The sometimes adversarial nature of data collection leads to several challenges:

–* Many social platforms discourage data collection by third parties*. Social media platforms may offer no programmatic access to their data, prompting researchers to use crawlers or “scrappers” of content, or may even actively discourage any type of data collection via legal disclaimers and technical counter-measures. The latter may include detection methods that block access to clients suspected of being automatic data collection agents, resulting in an escalation of covert (“stealth”) crawling methods (Pham et al., 2016). Platforms may also display different data to suspected data collectors, creating a gap between the data a crawler collects and what the platform shows to regular users (Gyongyi and Garcia-Molina, 2005).

–* Programmatic access often comes with limitations*. Some platforms provide Application Programmer Interfaces (APIs) to access data, but they set limitations on the quantity of data that can be collected within a given timeframe, and provide query languages of limited expressiveness (Morstatter et al., 2013; González-Bailón et al., 2014b; Olteanu et al., 2014a) (we discuss the latter in section 5.2). In general, legal and technical restrictions on API usage prevent third parties from collecting up-to-date, large, and/or comprehensive datasets. A main basis of these limits is probably that data is a valuable asset to these platforms, and having others copy large portions of it may reduce their competitive advantage.

–* The platform may not capture all relevant data*. Development efforts are naturally driven by the functionalities that are central to each platform. Hence, user traces are kept for the actions that are most relevant to the operation of a platform, such as posting a message or making a purchase. Other actions may not be recorded, e.g., to save development costs, minimize data storage costs, or even due to privacy-related concerns. For instance, we often know what people write, but not what they read, and we may know who clicked on or “liked” something, but not who read it or watched it (Tufekci, 2014). While these may seem to capture different behavioral cues, they can sometimes be used to answer the same question, e.g., both what people write and read can be used to measure their interest in a topic. Yet, using one or another may result in different conclusions.

–* Platforms may not give access to all the data they capture*. Some data collection APIs' restrictions stem from agreements between the platform and its users. For instance, social media datasets typically include only *public* content that has not been deleted, to which users have not explicitly forbidden access by setting their account as private, or to which users have given explicit access (e.g., through agreements or by accepting a social connection) (boyd and Crawford, 2012; Maddock et al., 2015).

–* Sampling strategies are often opaque*. Depending on the social platform, the available APIs for sampling data further limit what and how much of the public data we can collect; often offering few guarantees about the properties of the provided sample (Morstatter et al., 2013; Maddock et al., 2015). For instance, an API may return up to *k* elements matching a criteria, but not specify exactly how those *k* elements are selected (stating only that they are the “most relevant”). Further, in the case of Twitter, much research relies on APIs that give access to at most 1% of the data (González-Bailón et al., 2014a; Joseph et al., 2014; Morstatter et al., 2014). Data from these APIs have been compared against the full data stream, finding statistical disparities (Yates et al., 2016).

### 5.2. Data Querying

Data access through APIs usually involves a *query* specifying a set of criteria for selecting, ranking, and returning the data being requested. Different APIs may support different types of queries.

**Common issues**. There are a number of challenges related to the formulation of these queries:

–* APIs have limited expressiveness regarding information needs*. Many APIs support various types of predicates to query data, such as geographical locations/regions, keywords, temporal intervals, or users; and the combination of possible predicates determines their expressiveness (González-Bailón et al., 2014a). The specific information required for a particular task, however, might not be expressible within a specific API; which may result in data loss and/or bias in the resulting dataset.

For instance, keywords-based sampling may over-represent content by traditional media (Olteanu et al., 2014a) or content posted by social-media literate users, while geo-based samples may be biased toward users in large cities (Malik et al., 2015). Further, not all relevant content necessarily includes the chosen keywords (Olteanu et al., 2014a) and not all relevant content might be geo-tagged.^15^

–* Information needs may be operationalized (formulated) in different ways*. The operationalization of information needs in a query language is known as *query formulation*. There may be multiple possible formulations for a given information need, and distinct query formulations may lead to different results.

For instance, in *location-based data collections*, different strategies to match locations, such as using message geo-tags or the author's self-declared location, affect the user demographics and contents of a sample (Pavalanathan and Eisenstein, 2015). Studies relying on geo-tagged tweets often assume that geo-tags “correspond closely with the general home locations of its contributors;” yet, Johnson et al. (2016) found this assumption holds only in about 75% of cases in a study of three social platforms.

In *user-based data collections*, the selection criteria may include features held at a lower rate by members of certain groups (Barocas and Selbst, 2016), and it may over or under-emphasize certain categories of users such as those that are highly-active on a target topic (Cohen and Ruths, 2013). As a result, the proxy population represented the resulting dataset might fail to correctly capture the population of interest (Ruths and Pfeffer, 2014).

Query formulation strategies can also introduce *linking biases* (section 3.5), affecting the networks reconstructed from social media posts; query formulations may affect network properties (e.g., clustering, degree of correlation) more than API limitations (González-Bailón et al., 2014b).

–* The choice of keywords in keyword-based queries shapes the resulting datasets*. A recurrent discussion has been the problematic reliance on keyword-based sampling for building social media datasets (Magdy and Elsayed, 2014; Tufekci, 2014).

What holds for keywords holds for hashtags; plus, different hashtags used in the same context (e.g., during a political event) may be associated with distinct social, political or cultural frames, and, thus, samples built using them may embed different dimensions of the data (Tufekci, 2014). Ultimately, hashtags are a form of social tagging (or folksonomies), and even if we assume that all relevant data is tagged, their use is often inconsistent (varying formats, spellings or word ordering) (Potts et al., 2011).^16^ While some attempts to standardize the use of hashtags in certain contexts exist [e.g., see OCHA (2014) for humanitarian crises or Grasso and Crisci (2016) for weather warnings], hashtag-based collections may overlook actors that do not follow these standards.

There are efforts to improve and automatize data retrieval strategies to generate better queries (Ruiz et al., 2014)—including by expanding and adapting user queries (Magdy and Elsayed, 2014), by exploiting domain patterns for query generation and expansion (Olteanu et al., 2014a), by splitting the queries and run them in parallel (Sampson et al., 2015), or by employing active learning techniques (Li et al., 2014; Linder, 2017)—to mitigate possible biases by improving sampling completeness or representativeness.

### 5.3. Data Filtering

Data filtering entails the removal of irrelevant portions of the data, which sometimes cannot be done during data acquisition due to the limited expressiveness of an API or query language. The data filtering step at the end of a data collection pipeline is often called post-filtering, as it is done after the data has been acquired or obtained by querying (hence the prefix “post-”).

**Common issues**. Typically, the choice to remove certain data items implies an assumption that they are not relevant for a study. This is helpful when the assumption holds, and harmful when it does not.

–* Outliers are sometimes relevant for data analysis*. Outlier removal is a typical filtering step. A common example is to filter out inactive and/or unnaturally active accounts or users from a dataset. In the case of inactive accounts, Gong et al. (2015, 2016) found that a significant fraction of users, though interested in a given topic, choose to remain silent. Depending on the analysis task, there are implications to ignoring such users. Similarly, non-human accounts (discussed in section 4.4) often have anomalous content production behavior, but despite not being “normal” accounts, they can influence the behavior of “normal” users (Wagner et al., 2012), and filtering them out may hide important signals.

–* Text filtering operations may bound certain analyses*. A typical filtering step for text, including that extracted from social media, is the removal of functional words and stopwords. Even if such words might not be useful for certain analyses, for other applications they may embed useful signals about e.g., authorship and/or emotional states (Pennebaker et al., 2003; Saif et al., 2014), threatening as a result the research validity (Denny and Spirling, 2016).

## 6. Issues Introduced while Processing Data

**Definition (Data processing biases)**. Biases introduced by data processing operations such as cleaning, enrichment, and aggregation.

Assumptions in the design of data processing pipelines can affect datasets, altering their content, structure, organization, or representation (Barocas and Selbst, 2016; Poirier, 2018). Biases and errors might be introduced by operations such as cleaning (section 6.1), enrichment via manual or automatic procedures (section 6.2), and aggregation (section 6.3).

**Implications**. Bad data processing choices are particularly likely to compromise the internal validity of research, but they may also affect the ecological/external validity. For example, crowdsourcing is one of the dominant mechanisms to enrich data and build “ground truth” or “gold standard” datasets, which can then be used for a variety of modeling or learning tasks. However, some “gold standards” have been found to vary depending on who is doing the annotation, and this, in turn, may affect the algorithmic performance (Sen et al., 2015). As a result, they can affect both type I and II research (section 2.1).

### 6.1. Data Cleaning Issues

The purpose of data cleaning is to ensure that data faithfully represents the phenomenon being studied (e.g., to ensure construct validity). It aims to detect and correct errors and inconsistencies in the data, typically until “cleaned” data can pass consistency or validation tests (Rahm and Do, 2000). Data cleaning is not synonym for data filtering: while data cleaning may involve the removal of certain data elements, it can encompass data normalization by correction or substitution of incomplete or missing values.

**Common issues**. Data cleaning procedures can embed the scientist's beliefs about a phenomenon and the broader system into the dataset. While well-founded alterations improve a dataset's validity, data cleaning can also result in incorrect or misleading data patterns, for example:

–* Data representation choices and default values may introduce biases*. Data cleaning involves mapping items, possibly from distinct data sources, to a common representation.^17^ Such mappings may introduce subtle biases that affect the analysis results. For instance, if a social media platform allows “text” and “image” postings, interpreting that an image posting without accompanying text has (i) null text, or (ii) text of zero length, can yield different results when computing the average text length.

–* The normalization of text or geographical references may introduce biases*. We noted that geographical references in social data can be problematic (see also section 5.2). Users of some social platforms have various choices for geographically annotating profiles and content. Cleaning may involve replacing missing values or making estimations to geo-locate objects within a location at a given geographical granularity (e.g., city or country level). This may introduce errors, for instance, by mapping a description of a location to the coordinates of the center of the geographical bounding box containing the given location.^18^

Similarly, common text normalization steps such as lowercasing, spell corrections, removing word inflections by mapping it to the base form (i.e., lemmatization), or pruning words down to their stems (i.e., stemming), can also introduce errors and skew results (Denny and Spirling, 2016). Such procedures can collapse terms with different meanings under the same representation e.g., lowercasing the name “Iris” will make it indistinguishable from the flower name, while stemming the terms “[tree] leaves” and “[he is] leaving” will lead to an identical representation, “leav.”

### 6.2. Data Enrichment

Data enrichment involves adding annotations to data items that can be used during the analysis phase. Annotations may range from simple categorical labels associated to each item, to more complex processing such as part-of-speech tagging or dependency parsing done on text. They can be obtained through either some form of (semi-)automatic classification, or through human-annotations (e.g., crowdsourcing, surveys).

**Common issues**. However, both manual and automatic annotation are prone to errors (Cohen and Ruths, 2013), and can both exacerbate existing biases, as well as introduce new biases and errors.

–* Manual annotation may yield subjective and noisy labels*. Many factors affect the quality of human-annotations, including: (i) unreliable annotators, (ii) poorly specified annotation tasks and guidelines, (iii) poor category design (categories that are too broad, too narrow, or too vague), or (iv) insufficient information to make a reliable assessment (Cheng and Cosley, 2013; Joseph et al., 2017). Though the goal of an assessment task is to provide human input, underspecification or appeal to subjective judgment can introduce unintended biases that are often hard to detect. In fact, for many annotations tasks, the characteristics of those that do the annotations can significantly influence how they annotate (Olteanu et al., 2017a; Patton et al., 2019).

Further, certain annotation categories or attributes may be more easily recognizable than others. An example here may be the annotation of user profiles with demographic features. From inspecting a user profile, an annotator may be more likely to correctly identify a user gender than their age (Nguyen et al., 2014), and some categories may be easier to identify than others (e.g., “baby” may be a category in which annotators make less errors than “in their early 50s”). Such gaps across categories or attributes may introduce systematic biases in the data.

–* Automatic annotation via statistical or machine learning methods introduces errors*. A wide range of automatic processes may be used to enrich data: text can be processed through a complex Natural Language Processing (NLP) pipeline; other elements can be annotated with specialized classifiers or other types of annotators. What these processes have in common is that they apply some type of statistical or machine learning techniques, which are almost never 100% accurate.

For instance, automatic classification, a common operation of this kind, can introduce biases in the data. This is particularly problematic when the end goal is not the estimation of specific labels, but measuring their prevalence in the data [e.g., Gao and Sebastiani (2016) discuss why the distinction between the two tasks is important, and why different evaluation metrics should apply]. However, many social data analyses rely on machine learned classifiers to classify first, and count later (e.g., Mislove et al., 2011; Zagheni et al., 2014; Abbar et al., 2015).

In general, automatic classifiers used for data enrichment may not be robust across distinct datasets or not even across distinct classes of data within each dataset; for instance, it is easier to predict the political leaning of active users (Cohen and Ruths, 2013). The same observation holds when NLP tools for, e.g., language identification and dependency parsing are used to enhance textual messages; by focusing on African-American English dialect, Blodgett et al. (2016) show racial disparity when the textual content produced by users vary from the mainstream or standard languages.

### 6.3. Data Aggregation

Data aggregation is performed to structure, organize, represent or transform data; consider pre-processing heuristics that aggregate data to make it more manageable at the cost of losing information. Aggregation can also reduce or increase the prominence of distinct patterns (Olteanu et al., 2014b;Poirier, 2018).

**Common issues**. How these aggregations are done, or what information they compromise may lead to different conclusions. When aggregating geographically, one can indeed engage in a form of “gerrymandering” leading to vastly different results.^19^ When considering the overall incidence of distinct topics across users, aggregating contents by user may give equal weight to each user's interests, while aggregating by topic may give more weight to the content from highly active users. Furthermore, if the data is organized along a certain attribute (e.g., the presence of a keyword or hashtag), and there are multiple independent factors that result in the attribute taking a certain value, analyzing data entries with this value is equivalent to conditioning on it, and may result in spurious patterns of association among these factors (Blyth, 1972; Tufekci, 2014).

## 7. Methodological Pitfalls When Analyzing the Data

The choice of an analysis methodology typically reflects a researcher's experience and perspective, and may generate various concerns, such as (i) *using data as a source of hypotheses* rather than a tool to test them; (ii) *tailoring the research agenda based on data availability*, which can result in bias in the type of questions being asked; or (iii) *testing multiple hypotheses* until a significant, positive result is found. The latter includes practices such as *feature hunting* (Ruths and Pfeffer, 2014): greedily testing multiple features for classification tasks until finding the one that delivers the best improvements, instead of selecting these features based on *a priori* hypotheses.

**Implications**. An important consequence here is lack of replicability. For instance, due to variations in the analysis methodology, measurement, and data collection, Liang and Fu (2015) could not replicate 6 out of 10 known propositions from social media studies. More generally, the internal and external validity of both type I and type II research may be affected by the choice of what methods to apply when analyzing the data in order to characterize user populations and behaviors (sections 7.1–7.2), to make inferences and predictions (section 7.3), and to distill (causal) relationships (section 7.4).

### 7.1. Qualitative Analyses

Qualitative analyses tend to be in-depth, open-ended, and exploratory, answering questions about the *how, what*, or *why* of a social phenomenon. In comparison to quantitative methods, they tend to be based on smaller data samples (hence they are sometimes dubbed “small-N” methods).^20^ While the availability of large social datasets makes them suitable for quantitatively depicting behavior and populations, qualitative analyses are also used in social data research (boyd et al., 2010; Marwick, 2014; Tufekci, 2014), either alone or in conjunction with quantitative methods.

Qualitative analyses help construct hypotheses about phenomena to be quantified (Charmaz, 2014); can be used for in-depth explorations of quantitative measurements to validate or discern the nuances of their social meanings (Cranshaw et al., 2012; Tufekci, 2014); or can involve the development of codebooks to quantitatively code larger corpora (Vieweg et al., 2010). For instance, in-depth user interviews may help to explore how social media usage affects social ties (Burke and Kraut, 2014).

**Common issues**. Qualitative methods, though rich and illuminating when performed in conjunction with quantitative methods (Creswell and Clark, 2011), have known limitations when used in isolation. They tend to compromise generalizability (or external validity) for details (Trochim and Donnelly, 2001), mainly due to their limited scope, such as limited sample size (Lampe et al., 2008) or time period (Burke and Kraut, 2014). They are also more sensitive to researchers' interpretation biases, challenging to scale, and resistant to quantification (e.g., we may learn through interviews that people sometimes share content they dislike, but we do not know how often they actually do this).

### 7.2. Descriptive Statistics

Descriptive analyses are the basis of many studies, quantitatively depicting social data through numerical or graphical summaries of variables of interest, such as geographical distribution of messages (Leetaru et al., 2013), or temporal associations among topics (Fourney et al., 2015). Such analyses capture the distribution, variability, or correlations among variables, such as Java et al. (2007)—one of the first studies to characterize the growth, topological, and geographical properties of Twitter using descriptive statistics.

**Common issues**. The act of summarizing complex datasets with a small number of measures may conceal important details, potentially leading researchers toward wrong conclusions by compromising the internal validity of research.

–* Social data research often relies on counting entities*. These entities can be users, links, or messages, and the description is a summary of these counts (Lazer et al., 2014; Salganik, 2017). Yet, simple counts can mislead if it is unclear what is counted and how. The finding was later refuted, as the increase was due to a repeated message coming from a single pager (Pury, 2011). Based on how and when a distinction is made between content created by users and content re-shared by them (e.g., tweets vs. retweets), such confusions may also occur in other studies, e.g., that looked at volume-based trends or at the use of language.

Count-based analyses are also sensitive to confounders and issues with construct validity. For instance, popular strategies to characterize the emotional state of users rely on counting affectively positive and negative terms; yet, Beasley and Mason (2015) indicate that term frequency is an imprecise measure for how users truly feel. Another example are neighborhood maps created based on the frequencies of co-visits, which identify different neighborhood boundaries when conditioning for possible confounding factors (Kıcıman et al., 2014). In addition, using the average to summarize a measure that follows a power law may lead to distortion; power laws in degree distributions of social networks may also lead to paradoxes such as the friendship paradox (Jackson, 2016) or the majority illusion (Lerman et al., 2016). This can exacerbate issues introduced when the objects being counted are obtained through an automatic classification approach (as we discussed in section (6.2).

–* Correlational analyses are sensitive to bias and confounders*. Many studies assume that co-occurring patterns reflect true relationships, a common task being the extraction of associations among dataset variables (e.g., sources and types of information Olteanu et al., 2015) or with “offline” variables (e.g., food mentions on social media and obesity rates Abbar et al., 2015).

Such assumptions are problematic when social data does not accurately capture target offline or online populations (Hargittai, 2007), or user behavior is distorted by online or offline phenomena (Ruths and Pfeffer, 2014; Olteanu et al., 2015) (see section 3). Many datasets are built around dependent variables (see section 5.2), deciding to include a user or a piece of content depending on the inclusion of a variable under analysis (Tufekci, 2014); this may result in apparent patterns of association that fail to generalize. The challenge is how to distinguish between attributes that merely correlate and those that are causally related. For instance, Liang and Fu (2015) show that previously discovered correlations among the URLs found in tweets and their retweet rates may be spuriously induced by URLs co-occurring often with hashtags.

### 7.3. Inferences and Predictions

Beyond social data use for descriptive purposes, many studies aim to draw conclusions beyond the dataset under analysis. They use smaller (more manageable) samples to make inferences about unseen or larger populations, or use historical known measurements to predict their current (“*nowcasting”*) or future (*forecasting*) values using social data (Asur and Huberman, 2010; Salganik, 2017).

**Common issues**. Performing inferences and predictions using social data have proved harder than early results suggest, with many reported pitfalls (particularly w.r.t. construct and external validity) around attempts to infer and extrapolate results regarding political orientation (Cohen and Ruths, 2013), users' mood (Beasley and Mason, 2015) or location (Jurgens et al., 2015b; Pavalanathan and Eisenstein, 2015), or exit pools or election results (Gayo-Avello et al., 2011; Gayo-Avello, 2012;Gayo-Avello, 2013).

–* There are performance variations across and within datasets*. Even a very accurate model may introduce systematic errors concentrated on certain classes of messages or of users (Cohen and Ruths, 2013; Pavalanathan and Eisenstein, 2015; Tramer et al., 2015). Indeed, several empirical studies show that the performance of existing inference models is sensitive to various user-related confounds such as age or gender (Pavalanathan and Eisenstein, 2015;Landeiro and Culotta, 2016).

User-related confounds are not the only culprits. For instance, Denny and Spirling (2016) shows that topic modeling techniques such as Latent Dirichlet Allocation (LDA)—frequently used in analyses of textual content created or shared by users—yield different results depending on the application of common pre-processing steps for textual data. Further, a dataset might just not capture sufficient information to make an inference; for example, there are limits to approaches for predicting users' demographics solely based on the messages they post in social media (Nguyen et al., 2014).

–* The composition of test and training data samples impacts the results*. (see, e.g., Cohen and Ruths, 2013; Nguyen et al., 2014; Jurgens et al., 2015b; Pavalanathan and Eisenstein, 2015). For instance, using data samples biased toward users whose gender (Rao et al., 2010) or political identity (Cohen and Ruths, 2013) are easy to discern, leads to overoptimistic performance estimations that do not reflect those obtained on balanced or representative samples) (Cohen and Ruths, 2013;Nguyen et al., 2014).

–* Distinct target variables, class labels, or data representations may lead to different results*. When dealing with “fuzzy” constructs for which there is no gold standard (see also sections 2.2 and 6.1), studies often end up using varying definitions and proxies for the target variable (e.g., political leaning) and class labels (e.g., democrats or republicans), leading to results that are hard to compare or generalize (Cohen and Ruths, 2013; Wong et al., 2013). Even for less ambiguous constructs (e.g., user location) there can be multiple competing proxies, whose choice can impact a study's results: e.g., the accuracy of text-based geo-location of Twitter users varies across samples, based on whether either the user-supplied location or their tweets' GPS coordinates were used as proxies for user location (Jurgens et al., 2015b; Pavalanathan and Eisenstein, 2015).

In general, the *data representation* or *features* selected to represent an object, such as a user or a message, impacts the results of inference tasks on those objects. For instance, even if a user sample is representative, some features may occur at lower rates in the messages of certain users (Gong et al., 2015). For a discussion of these issues beyond social data research see Barocas and Selbst (2016).

–* The choice of the objective function can misguide the inference task*. Risks are also linked to the selection of the objective functions used to express various inference or prediction tasks (Wagstaff, 2012); such as using a wrong objective function that does not match the inference methodology (Gao and Sebastiani, 2016), or one that leads to undesirable behavior during the learning process or that is expensive to reliably evaluate (Amodei et al., 2016). Similarly, at times a concrete objective function will only approximate the true objective. For example, in a web search scenario, the true objective criterion may be user satisfaction, but this is often approximated by behavioral signals such as clicks or query reformulations (White, 2016). Moreover, these surrogate objectives themselves might also be based on imprecise measurement or biased modeling (Mehrotra et al., 2017), and have the potential to create self-fulfilling feedback loops when decisions are made based on the inference results and the outcomes are fed back into the models as training data (Barocas, 2014).

### 7.4. Observational Studies

Many studies also aim to determine *why* something is happening; that is, causation. For this, a study would typically seek to compute the effect of a *treatment* or an *intervention* (e.g., receiving a recommendation) on users, systems, or phenomena. The gold standard for such causal analyses are randomized controlled experiments (Aral and Walker, 2011; Muchnik et al., 2013). When experimentation is impractical or unethical, researchers often resort to conducting observational studies with social data.

In addition to identifying natural experiments (where assignment to treatment is random or “as good as random”), there are methods that help assess causation in observational studies and mitigate the effects of confounding or selection bias, under strong assumptions; including matched analysis (De Choudhury et al., 2016; Sharma and Cosley, 2016), instrumental variables analysis (Sharma et al., 2015), regression discontinuities (Malik and Pfeffer, 2016), and differences-in-differences (Carmi et al., 2012;Zagheni et al., 2014).^21^

**Common issues**. Unfortunately, while determining causality through active experimentation is already difficult, observational studies can be even more challenging due to the difficulty in accounting for the effects of uncontrolled confounds. With all of these methods there are critical caveats and strong assumptions that must be accounted for; otherwise, they are also susceptible to various validity issues (Oktay et al., 2010). Challenges for observational studies through social data include:

–* Social data may not capture the entirety of users' lives*. A key assumption of observational studies is that all covariates that affect treatment status and outcomes are observed, and that unobserved covariates are *ignorable*. However, it is possible that some unobserved covariates such as environmental factors or individual characteristics and actions may in fact affect users' propensity to be treated, as well as their eventual outcomes. Without significant domain expertise this assumption is often hard to fully assert.

For instance, network studies of peer influence and social contagion suffer from a stubborn challenge of disambiguating such effects from homophily among peers and within communities (Christakis and Fowler, 2007; Lyons, 2011; Shalizi and Thomas, 2011). While Christakis and Fowler (2007) found obesity to spread through peer influence in social networks, others suggest that unobserved confounds correlating with the social network structure (Lyons, 2011) or environmental exposure (Cohen-Cole and Fletcher, 2008) may be the culprit rather than peer influence.

–* Peer effects due to platform affordances and conventions may weaken causal analyses*. Another key assumption of many analyses is that the effect of a treatment on an individual is independent of the treatment status of others. Alas, this assumption is often violated in the presence of network effects, including common social features (e.g., hashtags, messaging, community support) that provide value through network usage (Ugander et al., 2013). For example, a conversation on a topic may include (re)shared content or hashtags and, thus, one user's use of a term may have an effect, sometimes called *network interference*, on the utility observed by others in an online community (Eckles et al., 2017; Olteanu et al., 2017b).

–* The identification of (non-)treated users may pose internal and construct validity threats*. Social media studies often rely on self-reports to identify treated users by searching for certain terms in messages (Proserpio et al., 2016; Olteanu et al., 2017b), e.g., identifying job losses by searching for statements such as “I was fired.” However, not all treated users will report their treatments, and some identified reports may be untruthful or inaccurate (Proserpio et al., 2016).

Further, to identify a *control* group—used as baseline in causal analyses—studies employ various sampling strategies including random sampling, network based sampling (e.g., friends or followers), or topical or domain based sampling (e.g., select users taking different drugs than the one under analysis) to identify similar users with those treated, but that have not received the treatment (Pavalanathan and Eisenstein, 2016; Olteanu et al., 2017b). Yet, different strategies may lead to different degrees of similarity among the treated users and the control group, and, thus, to different estimates of the treatment effect (Oktay et al., 2010).

–* Selection bias and how treatment effects are estimated affect result generalizability*. First, many methods compute only the local average treatment effects for a selected (sub)population (Nichols, 2007), limiting the generalizability of results to users with different characteristics than those included in a study. This is important for social data studies that typically suffer from self-reporting biases (as mentioned above and in section 3.3), and are thus limited to the association patterns captured by each working dataset (De Choudhury et al., 2016; Olteanu et al., 2017b).

Second, there can also be heterogeneity in the effects of a treatment across users, and, as a result, the average treatment effect (even when calculated under sound assumptions or for randomized experiments) may not generalize to all treated users (Taylor et al., 2014).

## 8. Issues With the Evaluation and Interpretation of Findings

A last opportunity to account for biases and gauge the reliability of findings is when evaluating and interpreting a tool performance or the results of a study. A good starting point is a proper understanding of the nature of the data being used. For instance, Rost et al. (2013) argue that data explicitly generated by users on social media should, in fact, be interpreted as communicative rather than representative, as these data are often a record of communication instead of a direct representation of other (“real-world”) types of behavior; raising questions about construct and internal validity.

**Implications**. How the evaluation is performed in a study may lead to biased conclusions or outcomes, including due to metrics selection (section 8.1) or results assessment and interpretation (section 8.2), which can both pose threats to construct validity. Such issues can also raise concerns about the reproducibility of a study; and when biases are not accounted for, failing to properly acknowledge potential limitations (section 8.3) may conceal important validity issues that may affect both type I and type II research.

### 8.1. Metrics Selection

Metrics are used to quantify a phenomenon (e.g., popularity or interest), or to measure the performance of some method or tool.

**Common issues**. When working with social data, the metrics employed in a study are often only proxies for some values of interest (e.g., sharing patterns as a proxy for popularity)—sometimes corresponding to latent or unobserved constructs. As a result, these metrics may be inconclusive, or they may suffer from reliability and construct validity issues.

–* The choice of metrics shapes a research study take-aways*. Metrics may attempt to quantify the relationship between the design or actions of a system and an outcome. For example, the effectiveness of a web search engine might be quantified by the click-through rate on the search results page. However, two metrics aimed at measuring the same aspect (e.g., user satisfaction) may be inconsistent with one another depending on the context. Further, Olteanu et al. (2014b) and Jurgens et al. (2015b) study social media and recommendations respectively, and review how computing a metric (e.g., precision) in a user-centric vs. an inference-centric fashion may lead to different measurements (see section 6.3). The latter may be biased toward the most active users, and may obscure the distribution of this metric across users.

–* Assessing fairness comes with its' own challenges*. In general, result metrics are aggregates and, thus, sensitive to the way in which the aggregation is done (see section 6.3). In the case of metrics of individuals, these aggregations can obscure manifestations of deeper structural inequity (boyd and Crawford, 2012; Barocas and Selbst, 2016). Researchers have also developed a growing body of evaluation metrics for measuring fairness (Dwork et al., 2012; Kearns et al., 2017; Heidari et al., 2018; Verma and Rubin, 2018). These fairness metrics are often centered around and, thus, bounded by practitioners' ability to define a task specific similarity metric for individuals (for *individual fairness* metrics) or to define a task specific error metric for groups (for *group fairness* metrics) (Dwork et al., 2012; Narayanan, 2018). Practitioners should be careful when adopting fairness metrics as each technical definition carry strong values assumptions which can often be in tension with each other (Friedler et al., 2016; Kleinberg et al., 2017).

–* Context- or domain-specific performance indicators are rarely used*. Systems using social data have just begun to be used in large-scale real-world applications; example domains include humanitarian response (Meier, 2015) and stock trading (Dredze et al., 2016). While preliminary reports may highlight the positive aspects of these deployments, there is a lack of rigorous longitudinal evaluations of the contribution of social data to improve domain-relevant metrics, such as “dollars saved, lives preserved, time conserved, effort reduced, quality of living increased” (Wagstaff, 2012).

Wagstaff (2012) and Rudin and Wagstaff (2014) raise concerns about the pervasiveness of abstract metrics, such as precision and recall, which explicitly ignore relevant domain-specific ones. Abstract metrics enable comparisons across domains, but offer limited insights about the actual improvements for each problem domain. For instance, perhaps 90% precision is appropriate for some applications (e.g., identify cat pictures for an image search engine), but not for others (e.g., identify criminal activities for law enforcement). Even when a metric indicates a good overall performance on a classification task, it is hard to know what that implies (Hardt, 2014), as errors may be concentrated in one particular class or group of classes (Konstan and Riedl, 2012;Hardt, 2014).

There are also questions about the stability and validity of abstract metrics (Sokolova and Lapalme, 2009). In social media research, the number of posts has been used as a proxy metric for the interest in a topic (Chen et al., 2010); yet, while this number may reflect production patterns, it may not reflect how much content on the topic users read (as seen in section 5.1). In the context of detecting hate speech online, Olteanu et al. (2017a) found that even when a given performance metric is fixed (e.g., precision), user perceptions of the output quality may vary based on various user characteristics. Finally, in some cases, metrics may themselves be designed using a statistical model, subject to the same biases presented in section 7.3 (Diaz, 2016).

### 8.2. Assessment and Interpretation of Results

A researcher own biases, perspectives and experience may be reflected in the way in which a system performance or an analysis' results are assessed and interpreted (Croskerry, 2002), and may also be dependent on the assumptions made about the data and the methods that were used.

**Common issues**. Much research rests upon the assumption that online social traces reflect in some quantifiable way real-world phenomena (Asur and Huberman, 2010; Kıcıman and Richardson, 2015), an assumption that is key when assessing and interpreting results. However, this assumption has been challenged due to concerns with construct validity and stability over time (Freelon, 2014; Lazer, 2015). Further, the particular choice of what methodological approach to use, as well as the datasets analyzed or used for training and testing purposes may also raise concerns about the internal and external validity.

–* The meaning of social traces may change with context; yet, this is hard to discern at evaluation time*. Rarely will a social network reflect homogeneous relations between individuals. Social links between users can stem from friendship, trust or shared interests, and thus can embed different social cues (Tang et al., 2012). Likewise, sharing content can be a sign of endorsement or interest, but users may also share content to ridicule, disapprove, or bully. The same mechanism or process may capture different signals across contexts (Tufekci, 2014), but such distinctions are often unintelligible and hard to make in an automated fashion when looking at a system output or at an analysis results in aggregate (Rost et al., 2013; Tufekci, 2014).

This unintelligibility is also subject to functional biases (section 4.1), as it depends on the mechanisms available on each social platform (e.g., having a like button, but not a dislike one), as well as to variations in platform algorithms and mechanisms in response to users actions (Lazer et al., 2014). It is difficult to properly account for what was or not in the data when researchers lack proper context (boyd and Crawford, 2012)—e.g., for social media use in crises, it may be hard for a geographically distant researcher to fully gauge the cultural context and the event specifics (Crawford and Finn, 2014). Distinct methodological alternatives may also lead to varying interpretations of what is in the data (Bruns, 2013), and thus those of the patterns drawn from it.

–* Analyses and evaluations confined to a single dataset or method may not generalize*. The confinement of many studies to a dataset or analysis method raise concerns about how much they generalize beyond particular setups, prompting calls for more comprehensive studies (Fraustino et al., 2012; Ruths and Pfeffer, 2014) (see section 3.3). Results of various methods to collect, measure, or process data should be routinely juxtaposed (Ruths and Pfeffer, 2014; Tufekci, 2014). When biases cannot be ruled out as the biasing factors are too complex or hard to untangle, running longitudinal, multi-datasets, cross-domain or platform analyses may be needed (Shani and Gunawardana, 2011; Schoen et al., 2013; Ruths and Pfeffer, 2014).

If access to multiple datasets is limited, the analysis can be run on datasets altered to introduce or remove noise or biases (Ruths and Pfeffer, 2014). Alternatively, general patterns can be probed across different classes of data (Bobadilla et al., 2013; Cohen and Ruths, 2013), as important variations may exist not only across datasets, but also within datasets due to differences in the demographics of users (Cohen and Ruths, 2013) or the types of items (Olteanu et al., 2014b).

–* The interpretation and assessment of results are too often done by data experts, not by domain experts*. This is problematic as there are known differences in how non-experts and experts interact with and validate systems outputs (White et al., 2009; Patton et al., 2019), particularly for critical application domains such as health. Furthermore, to interpret, e.g., the relations found by causal inference techniques as causal, among others, unobserved covariates are assumed ignorable; yet, without significant domain expertise this cannot be asserted (see section 7.4).

### 8.3. Disclaimers and Reproducibility

Finally, to foster reproducibility, there is a need to develop baselines and guidelines (Tufekci, 2014; Weller and Kinder-Kurlanda, 2015), to find common ground regarding methodological approaches (Counts et al., 2014), and to better document home-grown tools and methodologies, as well as data provenance (Bruns, 2013; Weller and Kinder-Kurlanda, 2015).

**Common issues**. While the natural language processing and information retrieval communities have developed a series of standard evaluation procedures and metrics, for many social media analysis tasks more effort is required to develop standardized experimental methodologies (Bruns, 2013;Diaz, 2014).

–* Disclaimers and negative results are overlooked*. While failed studies or negative results are useful to learn about what hypotheses were rejected, or what datasets or methods were found not suitable for a given problem, publications of negative results are scant (Gayo-Avello, 2012; Ruths and Pfeffer, 2014). There is an unfortunate bias against the publication of negative results (Fanelli, 2012)—e.g., describing failures to reproduce an existing result, approaches that did not deliver the expected results, like the features that did not improve a classifier performance, or algorithms that failed to deliver an acceptable performance.

In addition, disclaimers about the limitations of an analysis are fundamental to good practice. If errors or biases cannot be ruled out, researchers must discuss the gaps and limitations in their working datasets, their methods and their assumptions (Crawford and Finn, 2014; Ruths and Pfeffer, 2014; Tufekci, 2014). The risk of ambiguous generalizability claims should be considered, and the assumptions under which the results would hold to other context (e.g., other domains, platforms or populations) should be clarified.

–* There is a need to ease the task of sharing data and tools*. These are cornerstone for the reproducibility and replicability of studies.^22^ Being able to reproduce a given study, in fact, is primarily dependent on its external validity—i.e., on the ability to generalize the findings beyond the particular settings of that study—and, thus, particularly important for Type II research. Data sharing and tool sharing are needed to precisely evaluate and interpret research outcomes.

*Data sharing* may consist of providing datasets, or the details (including source code) for gathering exactly or approximately the same datasets when data sharing is prohibited by terms of service or privacy constrains. It can reduce redundant, labor-intensive, and time-consuming data collection, making social data research more inclusive and narrowing existing data access gaps (Jurgens et al., 2015a; Weller and Kinder-Kurlanda, 2015). Yet, Hutton and Henderson (2015b)'s study of 505 papers mentioning a social network between 2011-2013 revealed that only about 6% shared any data, while Zimmer and Proferes (2014) found that of 382 Twitter studies only about 5% use existing datasets collected by other researchers.

*Tools sharing* may include providing details (including source code) for understanding or executing an algorithm or for analyzing data. Beyond aiding reproducibility and future comparisons, the availability of tools may also enable the participation of those lacking the resources to create their own (e.g., many researchers outside of computer science) (boyd and Crawford, 2012; Bruns and Liang, 2012). Alas, releasing and maintaining code and tools is a laborious, non-trivial task and many researchers lack the incentives to do so.

## 9. Ethical considerations

Previous sections can be seen as covering what are ultimately ethical issues that Mittelstadt et al. (2016) calls *epistemic concerns* (sections 3–8), such as using evidence that is inconclusive or misguided. In contrast, this section deals with *normative concerns*, related mostly to the consequences of research.

Research on human subjects is regulated by law in many jurisdictions; and given that data elements in social datasets represent people or groups of people (Varshney, 2015; Diaz, 2016), research on social data is, arguably, human subjects research (Metcalf and Crawford, 2016). The fact that social data is often publicly accessible does not mean research done on it is ethical (Zimmer, 2010; boyd and Crawford, 2012). As a result, both scientists (Dwork and Mulligan, 2013; Barocas and Selbst, 2016) and journalists (Hill, 2014; Kirchner, 2015) have pressed for greater scrutiny of the use of social data against possible ethical pitfalls, such as breaching users privacy (Goroff, 2015), or enabling racial, socioeconomic or gender-based profiling (Barocas and Selbst, 2016).

Such ethical issues have been further highlighted by recent cases, including the Facebook contagion experiment (performed in early 2012 and published in late 2014), where researchers manipulated users' social feeds to include more or less of certain kinds of content based on the expressed emotions (Kramer et al., 2014). The experiment was criticized as an intervention that affected the emotional state of unsuspecting users, who had not given consent to participate in the study (Hutton and Henderson, 2015a). Another example is the Encore research project and how it measured web censorship around the world by instructing web browsers to attempt downloads of sensitive web content without users' knowledge or consent (Burnett and Feamster, 2015), potentially putting people in some countries at risk of harm due to these attempted accesses. In an unprecedented move, the Program Committee (PC) of SIGCOMM 2015 decided to accept the Encore research paper on the condition of placing a prominent note at the top of the paper highlight the PC's ethical concerns (Narayanan and Zevenbergen, 2015).^23^

The next section (section 9.1) depicts a key tension in research ethics of digital data. We then organize the discussion on specific ethical problems in social data research with respect to three basic criteria brought forward in the Belmont report (Ryan et al., 1978), a seminal work on research ethics; autonomy (section 9.2), beneficence (section 9.3) and justice (section 9.4).^24^ Given that our treatment of the subject is purposefully schematic, the interested reader can find more information in related works by Grimmelmann (2015),Metcalf and Crawford (2016), Bowser and Tsai (2015), Benton et al. (2017), and Mittelstadt et al. (2016).

### 9.1. Navigating a Fine Line: Research Ethics of Digital Data

Navigating ethical issues around social data requires reconciling two extreme perspectives: 1) social data research is similar to clinical trials and other human experiments in its capacity to harm people, and thus should be regulated as such; and 2) social data research is similar to other computing research, traditionally focused on methods, algorithms and system-building, with minimal direct impact on people.

**Social data research is different from clinical trials**. Many of the traditional processes to ensure ethical compliance in human subject research were developed in the context of clinical trials, which involve testing the effect of treatments on actual patients. These may have harmful, sometimes severe and irreversible unexpected effects. In contrast, the harm that common types of social data research can produce is often of a different nature, such as suffering a breach of privacy, or being exposed to disturbing images. An ethics approval process designed specifically for social data research, like the one brought forward by Bowser and Tsai (2015), which includes questions that are social media specific, or the set of practices outlined by Benton et al. (2017) could be more appropriate to decide whether a research activity should take place or not, and under what conditions.

**Ethical choices in social data research require deliberation**. Ethical choices are difficult because, among other reasons, they often involve several values that might be in conflict. For instance, data analysis may be needed to provide important services, and solutions that balance between privacy and accuracy should be considered (Goroff, 2015). In other cases, experimentation may be needed to determine which policies or treatments are appropriate—yet, Meyer et al. (2019) found an aversion to experiments, with people approving of untested policies being universally implemented but disapproving of randomized experiment to test which policy is better.

Computing professionals have varying degrees of preparation when it comes to addressing these kinds of problems. As a general rule, ethical issues are best addressed through informed deliberation and conversation. This is why approval and monitoring of research by Institutional Review Boards (IRBs) are important. IRBs set common standards within an institution, provide researchers with a framework to think critically about consequences, and show to others that careful decisions have been made for a study.

### 9.2. Respect to Individual Autonomy

Respect for the capacity of individuals to make autonomous decisions is often expressed in research through informed consent. Informed consent requires that (i) researchers *disclose* all relevant information to potential participants; (ii) potential participants are *capable* of evaluating this information; (iii) potential participants can *voluntarily* decide to participate or not; (iv) participants give researchers explicit *permission*, often in writing; and (i) participants are free to *withdraw* their consent at any point.

**Common issues**. Social data research poses particular challenges to the practice of informed consent:

–* Obtaining consent from millions of users is impractical*. Studies that leverage data from millions of social media users often do it without any kind of consent from them (Zimmer, 2010; Hutton and Henderson, 2015a). User data may have been provided freely online for anyone to access it, but it is inherently sensitive as users might not anticipate a particular use of their data, especially when created in a context-sensitive space and time (boyd and Crawford, 2012). This is even more delicate when analyzing user demographic attributes (Chou, 2015). While asking consent might be often seen as impractical (boyd and Crawford, 2012), there are efforts to design methodologies for acquiring consent while minimizing the burden on participants (Hutton and Henderson, 2015a).

–* Publicly sharing content online may not imply consent for research*. Even if we were to accept the notion that by placing their information in online public spaces, user consent for research is implied, “people's privacy preferences depend on their circumstances” (Crawford and Finn, 2014); and these preferences may or may not be reflected in privacy settings, which users rarely change (Wang et al., 2011). Take the case of social media use in crisis situations by vulnerable populations, which may publicly share personal information to assist others or ask for help. Such disclosures are closely coupled with their context, hence, the usage and sharing of data should be extensively scrutinized, and the privacy of these users should be protected outside the original context (Crawford and Finn, 2014).

–* Social platforms terms of use may not constitute informed consent for research*. By signing up for a social media platform, users accept their terms of use, which often contain blanket clauses allowing research for various purposes. The acceptance of terms of use may not fulfill the criteria of informed consent, as the often vague language alluding to “research use” does not involve a disclosure of the *specific* elements relevant to a particular research program. For instance, the aftermath of the Facebook emotional contagion experiment (Kramer et al., 2014) suggests that users were not aware of the risks or benefits of this research. Even if experiments were described clearly in a specific informed consent form for this type of experiment, the intimate nature of social platforms may require ongoing, dynamic consent, as is found in disciplines such as ethnography (American Anthropological Association, 2004).

### 9.3. Beneficence and Non-maleficence

Another key ethical criteria is concerned with the assessment of risks and benefits; specifically, research should be beneficial and not cause harm (non-maleficence). The researchers should deliberate over not only the benefits of research, but also over the possible types of harms (Barocas et al., 2017), the affected groups, and how to test for adverse impact (Sweeney, 2013).

**Common issues**. Research on social data is associated to specific types of harm, of which perhaps the most obvious are privacy breaches (Zimmer, 2010; Crawford and Finn, 2014).

–* Data about individuals can harm them if exposed*. Privacy breaches can have harmful outcomes (Barocas and Selbst, 2016) like stalking, discrimination, black-mailing or identity theft (Gross and Acquisti, 2005). Some prominent examples include the Ashley Madison data spill in 2015, where a site advertising itself as a dating network for cheating spouses had account information (including full names of users) stolen and posted online (Thomsen, 2015), as well as the more recent Facebook data spills where hundreds of million of records that include comments, likes, reactions, account names, app passwords, and more were publicly exposed.^25^

Further, archiving personal data for too long, or sharing poorly anonymized datasets publicly, contribute to privacy breaches as this data can be combined with other sources to gain insights about people without their knowledge (Crawford and Finn, 2014; Goroff, 2015; Horvitz and Mulligan, 2015). Prominent releases of anonymized data, such as for the Netflix prize and the AOL's Search History Database, were later found to provide inadequate protection to users (Barbaro et al., 2006; Narayanan and Shmatikov, 2008).^26^^,^^27^ If data is archived or shared, it should be processed, not only to remove obvious personal identifiers, but also to prevent re-identification via combinations of apparently non-sensitive attributes (Ohm, 2010).

–* Research outcomes may be used to do harm*. In addition to the fact that inferences drawn from social data may be incorrect in many ways, as this survey emphasizes, inferences that are too precise may create the capacity to finely discriminate among people into ever-smaller groups (Barocas, 2014). For instance, Matz et al. (2017) reflect on their (Kosinski and Stillwell) decade-long research program on infering personality traits from social media users and its potential for mass manipulation, as evidenced in the adoption of similar techniques in the manipulation of elections.^28^

–* “Dual-use” and secondary analyses are increasingly prevalent in social data research*. Thus, data, tools, and inferences obtained for one purpose can be used for another purpose (Hovy and Spruit, 2016; Benton et al., 2017); yet, the risks associated with secondary uses may not be well understood.^29^^,^^30^ The Cambridge Analytica's use of Facebook data also demonstrates how both the social data and the sentiment analysis techniques have dual-use: they can be used for ads targeting, as well as to tailor propaganda (Horowitz et al., 2018)^28^. Another example, protesters in Baltimore, USA, were arrested based on information gleaned from social media.^31^

### 9.4. Justice

An ideal of justice in research is that risks and benefits are justly apportioned, which requires to know at the onset who will be burdened by research, and who will benefit from the results.

**Common issues**. Key concerns include:

–* The digital divide may influence research design*. The *digital divide* is the gap that exists among and within countries or communities with respect to access to information and communication technologies. This gap has many manifestations, including the *data divide*: a lack of availability of high-quality data about developing countries and underprivileged communities (Cinnamon and Schuurman, 2013). Together, the digital divide and the data divide can be an important source of bias on the questions that are asked and the populations that are chosen for research (boyd and Crawford, 2012; Counts et al., 2014). They can focus the research agenda on so-called “first-world problems,” such as finding a restaurant, for which data is widely available.

–* Algorithms and research outcomes may lead to discrimination*. The reliance on automated decision making processes based on statistical methods, can inherit, propagate, or even amplify the biases and prejudice present in the training data with respect to various factors such as race, age, gender or socioeconomic groups (Crawford and Schultz, 2014; Barocas and Selbst, 2016). This problem is often referred to as *algorithmic discrimination* or *algorithmic bias* (see, e.g., Hajian et al., 2016).

–* Research outcomes may not be broadly available*. Providing users information about how their data are used is a key element concerning their autonomy (Horvitz and Mulligan, 2015). This transparency can also lead to a more just allocation of research benefits, yet this is rare. Ideally, people should have access to research results and artifacts that resulted from the study of their personal data (Gross and Acquisti, 2005; Crawford and Finn, 2014). Further, a failure to make data available may deepen the data divide (Bruns, 2013) and the gap between those that have the computational skills needed to analyze large volumes of data and those who lack them (boyd and Crawford, 2012; Weller and Kinder-Kurlanda, 2015).

–* Not all stakeholders are consulted about how research outcomes are being used*. Deliberations about how, for whom, and when to implement research outcomes should involve those that may be affected or whose data is being used (Costanza-Chock, 2018; Design Justice, 2018; Green, 2018)—following the “*nothing about us without us*” principle stating that no policy should be enacted without the direct participation of all affected stakeholders.^32^ This is often challenging as the way in which user data is processed and analyzed to support decision making tends to be “*black-boxed*” (Poirier, 2018). This may also be exacerbated by the rise of “embedded” researchers with privileged access to social platforms and ability to access data unavailable to broader groups (Crawford and Finn, 2014; Ruths and Pfeffer, 2014).

## 10. Discussion: Trends and Future Directions

There is a growing interest among researchers and practitioners to understand the limits of social datasets and social data methods; ethical challenges have also been brought to the forefront. At the same time, there are also more substantive discussions on how the lack of diversity among those that decide which research problems are being prioritized (e.g., through funding or peer review), as well as how, when, and for whom research outcomes are being implemented (Van Dijck, 2013a; Green, 2018; Hoffmann, 2018; West et al., 2019)—in other words, “*which humans are in the loop*” (West et al., 2019)—impact when and how issues with biases in the data, methods, or research outcomes are being addressed. We believe the need to identify, quantify, and address data biases, and methodological and ethical challenges around the use of social data, will remain a persistent and important issue for years to come.

However, eliminating all biases in social data is *unlikely*, perhaps even *undesirable*. Biases that bound the applicability of general solutions may help boost the performance of dedicated solutions (Yan et al., 2011; Olteanu et al., 2014a) or may inform their design (Olteanu and Pierre, 2012; Lerman and Hogg, 2014). Ultimately, as we stressed earlier (sections 1, 2), whether a research method or a dataset is adequate or not depends on the research question being asked, the context in which the research takes place, and, fundamentally, on the goals of the researcher(s).

In light of these trends, we expect the skepticism toward easy answers to continue to grow (section 10.1), as well as to see increasing efforts toward addressing these issues and developing standards and methodological best practices (section 10.2). We conclude with pointers to further readings (section 10.3).

### 10.1. A Trending Skepticism Toward Easy Answers

Following the well-known “hype cycle,” the phase of “inflated expectations” on social data research has perhaps already passed.^33^ We now recognize that the process by which social data are generated is more complex than what was once assumed, which translates into validity issues, meaning that the impact of various research studies might be more narrow than what was initially thought.

A growing number of research fora that critically examine computational and data driven research have emerged in disciplines that either focus on social data, or often use it. Newer venues include the ACM Conference on Fairness, Accountability, and Transparency (FAT*), the AAAI Conference on Artificial Intelligence, Ethics, and Society (AIES), along with several workshops, special issues in journals, and technical meetings. These venues often adopt an ethical framework based on fairness and transparency, to motivate discussions around the consequences of built-in biases in working datasets, and develop methodologies and ethical guidelines for the use of social data. Policies around these concerns may eventually emerge as the activity on the policy dimensions of these biases is increasing (Crawford et al., 2016; Goodman and Flaxman, 2016; US White House, 2016).

These efforts are embedded in a context of a broad reflection of common needs of computing research across the board, such as “the need for increasing awareness for what it is actually analyzed,” such as data and phenomena (Ruths and Pfeffer, 2014), or the need to understand various dimensions of the automated behavior of platform specific mechanisms such as their design and algorithms (Sandvig et al., 2014). In this context, the use of social data for both commercial and research purposes remains a core area of concern (boyd and Crawford, 2012; Sandvig et al., 2014; Salganik, 2017).

Moving forward, we expect a growing focus on three key areas that remain under-researched and not properly understood. First, while data biases are at times overlooked due to the personal blind spots of those working with social data (Holstein et al., 2019; West et al., 2019), a broader underlying issue, we argue, is *a persistent lack of understanding of how these data are created, what they actually contain, and how the working datasets are assembled* (sections 4–6): e.g., how and what is being logged? what can be logged or measured? how well do measurments approximate the phenomena of interest? how can we query or sample? who is (not) represented? As a result, how to properly collect or curate high-quality datasets, though critical in most application scenarios, also remains a lingering issue (Holstein et al., 2019).

Second, effectively identifying existing biases and other harmful blind spots along a data analysis pipeline further requires better auditing and evaluation frameworks, as well as metrics based on the semantics of the problem, rather than allowing them to be abstract or generic (Wagstaff, 2012). Users' perceptions and assessments of performance may also significantly diverge from that suggested by statistical metrics (Lee and Baykal, 2017; Olteanu et al., 2017a). In other words, *it is often unclear what is being evaluated* (section 8): e.g., is the performance or outcome of interest directly observable or measurable? are there competing proxies for the targeted performance or outcome of interest, and how reliable are they? how are the evaluation metrics aggregated across, e.g., users, regions, or behaviors?

Third, though increasingly pervasive, *the use of canned datasets and machine learning models is rarely scrutinized*. Many studies re-purpose existing datasets and pre-trained models for different uses, contexts or applications than those for which they were originally created. While in some cases they are central to a research study, in others their use is more subtle or peripheral: e.g., a study analyzing public opinion or popularity across demographics may infer those demographics using pre-trained models for facial recognition (e.g., Wang Y. et al., 2016b; Chakraborty et al., 2017). Gaps between the purpose and the assumptions under which these datasets and models are built and those under which they are being used can lead to performance disparities, affecting research validity and producing misleading results (e.g., Blodgett et al., 2016; Buolamwini and Gebru, 2018).

### 10.2. A Shift From Raising to Addressing Concerns About Social Data

For transparency and accountability, it is important to audit the social data as well as the algorithms and systems that manipulate them. In some cases biases can be hard to discover without a thorough, in-depth examination of a dataset or system.

With respect to data, a proposal of “Datasheets for Datasets” advanced by Gebru et al. (2018) suggests to maintain a careful registry of possible issues in data, including why and how it was collected and pre-processed, what are the policies for its re-distribution and maintenance, and outlining possible legal/ethical concerns. Similarly, others also suggest the use of such registries in the form of *model cards* that focus on documenting the creation of pre-trained models (Mitchell et al., 2019) or *supplier's declarations of conformity* to describe the lineage of AI services that can be “an amalgam of many models trained on many datasets” (Hind et al., 2018). Our **first recommendation** is to document in detail the process by which datasets and models are created, and to examine that process critically, including giving consideration to the biases we have described. Our **second recommendation** is to broaden studies on social data to different platforms, topics, timings, and sub-populations, to determine how results vary across, for example, different cultural, demographic, behavioral contexts.

Sandvig et al. (2014) argue that scrutiny is required even when a social software system appears to satisfy users' needs, as there can be “subtle patterns of problematic behavior” that are hard to discern. For instance, Kulshrestha et al. (2017) introduces a framework for auditing search systems on social media platforms by differentiating between various sources of bias (e.g., due to content or due to ranking algorithms). Audits sometimes require access to proprietary systems, which requires explicit permission to such systems, and that is likely to be denied if the goal is to expose or publicize their flaws. Reverse engineering these systems, or using them in an unanticipated way to expose their bias, may be illegal in the US under the Computer Fraud and Abuse Act (CFAA), which has been challenged in court by a group of researchers.^34^ Thus, our **third recommendation** is to enable transparency mechanisms that allow auditing social software and evaluating biases in social data at the source (section 4).

Further, there are also growing efforts to address social data limits, in the form of guidelines, standards, and new methodological approaches. These efforts include employing techniques from the causal inference literature that can lead to more robust research results (Landeiro and Culotta, 2016; Proserpio et al., 2016), or calibrating non-representative social data samples (Zagheni and Weber, 2015). Another direction is to employ standardized evaluation protocols when testing new tools or methodologies (Diaz, 2014; Jurgens et al., 2015b). Our **fourth recommendation** is to extend the research on these guidelines, standards, methodologies, and protocols, as well as to encourage their adoption.

Finally, given the complexities of the inherently contextual, application- and domain-dependent biases and issues in social data and analysis pipelines covered throughout this paper, there are no one-size-fits-all solutions—when assessing and addressing bias, *nuance, we argue, is critical*.

### 10.3. Further Reading

For additional discussions on the issues we cover in this survey, we recommend the books by Salganik (2017) and O'Neil (2016), talks by Wallach (2014) and Diaz (2016), and papers by Baeza-Yates (2018), boyd and Crawford (2012), Lazer and Radford (2017), Ekbia et al. (2015), Ruths and Pfeffer (2014), Tufekci (2014), Nguyen et al. (2016), and Barocas and Selbst (2016), among many others.

## Author Contributions

AO contributed conception of the review and initial writing. AO, CC, FD, and EK wrote sections of the manuscript. All authors contributed to manuscript revision, read and approved the submitted version.

### Conflict of Interest Statement

The authors declare that the research was conducted in the absence of any commercial or financial relationships that could be construed as a potential conflict of interest.

## Footnotes

^2^Patrick Tucker: “Refugee or Terrorist? IBM Thinks Its Software Has the Answer.” *Defense One*, January 2016. Online: http://www.defenseone.com/technology/2016/01/refugee-or-terrorist-ibm-thinks-its-software-has-answer/125484/ (accessed April 2019).

^3^Validity threats are particularly relevant when social data analysis is done to test hypotheses, but also relevant in e.g., predictive and exploratory projects.

^4^Dyslexia is a learning disorder characterized by difficulties in reading, spelling, writing, and/or pronouncing written words.

^5^See Clauset et al. (2009) for a discussion on when a heavy-tailed distribution is a power-low.

^6^E.g., for a discussion on the impact of Tweets length see: https://blog.twitter.com/en_us/topics/product/2017/Giving-you-more-characters-to-express-yourself.html (accessed April 2019).

^7^Pew Research Center: http://www.pewinternet.org/ (accessed April 2019).

^8^Tessa Harmon: “Stack Overflow's Developer Survey Analysis Hurts Women.” *Medium*, March 2018 Online: https://medium.com/@glitterwitch/stack-overflow-s-developer-survey-analysis-hurts-women-ec4d568e2352 (accessed April 2019).

^9^This definition of “behavioral bias” refers to a type of data bias. In cognitive psychology, “behavioral bias” has a different meaning: it is generally used to refer to the ways in which human decision making may systematically differ from the traditional rational account of decision making.

^10^As a phenomena, response bias was initially described in survey-based research in the psychology and sociology literature, e.g., see Gove and Geerken (1977); Randall and Fernandes (1991).

^11^From Facebook's Joel Seligstein, ICWSM'11 keynote, available at: http://videolectures.net/icwsm2011_seligstein_trends/ (accessed April 2019).

^12^LinkedIn is a social networking site oriented to professional usage, https://linkedin.com/

^13^Hawthorne effect. Wikipedia https://en.wikipedia.org/wiki/Hawthorne_effect (accessed April 2019).

^14^Danny Sullivan: ‘Google Bombs Aren't So Scary.” *Search Engine Watch*. May 2012. Online: https://www.clickz.com/google-bombs-arent-so-scary/61942/ (accessed April 2019).

^15^For instance, only 1%-2.9% on Twitter messages were geo-coded (Graham et al., 2014; Osborne and Dredze, 2014), 1% on Facebook and 0.6% in Google Plus (Osborne and Dredze, 2014).

^16^A form of *ad-hoc* categorization and labeling of the data within social systems (Specia and Motta, 2007).

^17^One example of this is Semantically Interlinked Online Communities (SIOC), an RDF standard for representing data from the social web: http://sioc-project.org/ (accessed April 2019).

^18^See https://medium.com/@ayman/the-social-concerns-of-geo-located-rectangles-9b361f34811d (accessed April 2019).

^19^A practice named after former US politician Elbridge Gerry, which consists of redrawing voting districts to favor a political party.

^20^Qualitative research is a complex methodological area. See, e.g., the textbook by Silverman (2013).

^21^For more background on causal inference with observational data, the interested reader can consult Nichols (2007).

^22^See Drummond (2009) for a discussion on the difference between reproducibility and replicability.

^23^SIGCOMM is a top-tier conference on computer networking.

^24^In the aftermath of World War II, the Nuremberg Code^25^ Available at http://www.cirp.org/library/ethics/nuremberg/. Accessed April 2019. provided the foundation for the development of human subjects research ethics, manifested in the Declaration of Helsinki (World Medical Association, 1964) and the Belmont report (Ryan et al., 1978). The latter outlines three ethical principles, autonomy: experiments should show respect for individuals; beneficence and non-maleficence: experiments should minimize risk for research participants and maximize benefits for society; and justice: risks and benefits of experiments should be fairly distributed.

^25^Third-party Facebook app data exposure, https://www.upguard.com/breaches/facebook-user-data-leak (accessed April 2019).

^26^Netflix Prize, https://en.wikipedia.org/wiki/Netflix_Prize accessed May 2019.

^27^AOL search data leak, https://en.wikipedia.org/wiki/AOL_search_data_leak (accessed May 2019).

^28^Edmund L. Andrews: “The Science Behind Cambridge Analytica: Does Psychological Profiling Work?” Stanford Graduate School of Business, April 2018. https://stanford.io/2HilsOg (accessed April 2019).

^29^This term has its' origin in the debate about the civil vs. military use of certain goods, http://ec.europa.eu/trade/import-and-export-rules/export-from-eu/dual-use-controls/index_en.htm (accessed April 2019).

^30^Ashley Crossman: “Pros and Cons of Secondary Data Analysis,” https://www.thoughtco.com/secondary-data-analysis-3026536 (accessed April 2019).

^31^“Facebook, Twitter, and Instagram surveillance tool was used to arrest Baltimore protesters.” The Verge, October 2016. http://www.theverge.com/2016/10/11/13243890/facebook-twitter-instagram-police-surveillance-geofeedia-api (accessed April 2019).

^32^“Nothing about us without us” principle, https://en.wikipedia.org/wiki/Nothing_About_Us_Without_Us (accessed April 2019).

^33^Gartner Hype Cycle, http://www.gartner.com/technology/research/methodologies/hype-cycle.jsp (accessed April 2019).

^34^Sandvig v. Lynch: Challenge to CFAA Prohibition on Uncovering Racial Discrimination Online. American Civil Liberties Union (ACLU), June 29th 2016. https://www.aclu.org/cases/sandvig-v-lynch-challenge-cfaa-prohibition-uncovering-racial-discrimination-online (accessed March 2017).
